# “Disruption of the molecular clock severely affects lipid metabolism in a hepatocellular carcinoma cell model”

**DOI:** 10.1016/j.jbc.2022.102551

**Published:** 2022-09-30

**Authors:** Natalia M. Monjes, Paula M. Wagner, Mario E. Guido

**Affiliations:** 1 CIQUIBIC-CONICET, Facultad de Ciencias Químicas, Universidad Nacional de Córdoba, Córdoba, Argentina; 2 Departamento de Química Biológica Ranwel Caputto, Facultad de Ciencias Químicas, Universidad Nacional de Córdoba, Córdoba, Argentina

**Keywords:** circadian rhythms, glycerophospholipid metabolism, clock genes, lipid droplets, metabolic oscillations, phosphatidylcholine, liver, HepG2, B-D, Bmal1-disrupted, B-WT, Bmal1-wildtype, CCT, phosphocholine cytidylyltranferase, CG, Clock gene, CCG, Clock-controlled gene, CHOK, choline kinase, CET: CTP, phosphoethanolamine cytidylyltranferase (coded by *Pcyt2* gene), DEX, dexamethasone, DG, diacylglycerol, DGAT2, glycerol-3-phosphate acyltransferase 2, FA, fatty acid, GPLs, glycerophospholipids, LD, lipid droplet, LAC, lactate, MG, monoacylglycerol, PC, phosphatidylcholine, PE, phosphatidylethanolamine, PI, phosphatidylinositol, PEMT, PE N-methyltransferase, PS, phosphatidylserine, ROR, retinoic acid orphan receptor, ROR, retinoic acid-related orphan receptor, ROS, reactive oxygen species, R/MO, redox / metabolic oscillator, TG, triglyceride

## Abstract

Involved in triglyceride (TG) and glycerophospholipid metabolism, the liver plays a crucial physiological role in the human body both as a major metabolic integrator and a central hub for lipid and energy homeostasis. Metabolic disorders can be caused by various factors that promote abnormal lipid accumulation in storage organelles called lipid droplets (LDs), as in hepatic steatosis, a metabolic syndrome manifestation that can progress to a hepatocellular carcinoma, the most common primary liver malignancy worldwide. Modern life involves conditions that disrupt the biological clock, causing metabolic disorders and higher cancer risk. A circadian clock is present in the liver and in immortalized cell lines and temporally regulates physiological processes by driving transcriptional and metabolic rhythms. Here we investigated metabolic rhythms in HepG2 cells, a human hepatocellular carcinoma–derived cell line, and the link between these rhythms and the circadian clock in control (Bmal1-wildtype) and *Bmal1*-disrupted (B-D) cells having their molecular clock impaired. Rhythms in the expression of lipid-synthesizing enzymes *ChoK*α, *Pcyt2*, and *Lipin1*, in the metabolism of particular glycerophospholipids such as phosphatidylcholine (PC) and phosphatidylethanolamine, and in the phosphatidylcholine/phosphatidylethanolamine ratio and TG and LD content were observed in Bmal1-wildtype cells. By contrast, in the B-D model, the whole hepatic metabolism was severely altered with a significant reduction in the TG and LD content as well as in *ChoK*α and other related lipid enzymes. Together, our results suggest a very strong crosstalk between the molecular clock and lipid metabolism, which exhibits an exacerbated pathological condition in B-D cells.

The liver is a major metabolic integrator crucial to physiological processes and constitutes a central hub for lipid metabolism, with uptake, esterification, oxidation, and secretion of fatty acids (FAs) occurring in hepatocytes. Fas in the liver originate in the diet, *de novo* lipogenesis, and/or recycling released from adipose tissue during fasting (1).

Glycerophospholipids (GPLs) containing FAs constitute an essential lipid family of biomembrane structural components exhibiting vital functions, such as cell signaling, energy balance, vesicular transport, cell division, and cell-to-cell communication, among others; they are also intermediate precursors of triglycerides (TGs) and components of lipid droplets (LDs). The most abundant GPLs present in all eukaryotic cells are phosphatidylcholine (PC) and phosphatidylethanolamine (PE), which are mainly biosynthesized through the “Kennedy and Weiss” *de novo* synthesis pathway (2). In the liver, the biosynthesis of PC may occur *via* the Kennedy pathway or through an alternative biosynthetic route in which the enzyme PE N-methyltransferase (PEMT) converts PE into PC (3). *De novo* PC synthesis involves three steps catalyzed by choline kinase (CHOK), CTP: phosphocholine cytidylyltranferase (CCT), and CDP-choline:1,2-diacylglycerol choline phosphotransferase. CCT activity has been considered the rate-limiting and regulatory step under most metabolic conditions (3). However, *ChoK* overexpression has been implicated in human carcinogenic processes (4, 5) and its regulation also influences PC biosynthesis as well as diacylglycerol (DG) availability (6, 7). *De novo* synthesis of PE mainly occurs from the CDP-ethanolamine Kennedy pathway. CDP-Etn formation is catalyzed by CTP: phosphoethanolamine cytidylyltranferase (CET codified by the *Pcyt2* gene) with Pcyt2 as the main regulatory enzyme [for a review see (8)]. In addition, phosphatidic acid, precursor of all GPLs, is dephosphorylated to DG by phosphatidate phosphohydrolase, which is codified by the gene *Lipin*1, to synthesize PC and PE or TGs. Changes in the PC and/or PE content of various tissues are implicated in metabolic disorders such as atherosclerosis, insulin resistance, and obesity. In the liver, abnormally high and low cellular PC/PE ratios influence energy metabolism and are linked to disease progression [see review in (9)]. LDs are dynamic organelles that during times of energy excess store neutral lipids in the core with a cover of GPLs and proteins, thus serving as an energy reservoir during deprivation (10). Specifically, changes in the hepatic PC/PE molar ratio have been linked to development of nonalcoholic fatty liver disease in humans, as well as in liver failure. Pathological conditions cause abnormal LD accumulation in the liver, so called obesity-related steatosis or nonalcoholic fatty liver disease which is hepatic manifestation of the metabolic syndrome and represents a huge public health problem (10, 11).

Modern life with hyper caloric diets, nocturnal shift work, prolonged artificial illumination all through day and night, etc. have severely altered the temporal organization of behavior and physiological processes. All living organisms have adapted through evolution to the day/night cycles, and most mammals have developed a circadian timing system to adjust their physiology and behavior to the 24 h light/dark cycle (12, 13, 14). This temporal organization relies on endogenous circadian clocks present in most tissues and organs and even in individual cells and is involved in the time regulation of essential cellular processes. Environmental or genetic disruption of circadian coordination causes metabolic imbalances, leading for instance to fatty liver, dyslipidemia, glucose intolerance, type II-diabetes, and obesity, thereby contributing to the development of a metabolic syndrome and a higher risk of cancer (15, 16, 17). Carcinogenesis is a complex and multietiological process resulting in the accumulation of genetic alterations primarily in genes involved in the regulation of signaling pathways relevant to the control of cell growth and division and deregulation of metabolism [reviewed in (18)]. Hepatocellular carcinoma (HCC) is the most common primary liver malignancy and is a leading cause of cancer-related death worldwide (19).

At the molecular level, the temporal organization of cells is driven by the molecular clock (see Fig. S1*A*) which displays a transcription–translation-based feedback loop. The core circadian oscillator is made up of a set of clock genes (CGs), involving activators such as *Clock* and *Bmal1* and repressor components like *Per1*, *Per2*, *Cry1*, and *Cry2* (20, 21). The transcription and translation cycle takes ∼24 h to be completed. A second alternative cycle involves CLOCK–BMAL1 complex that activates nuclear receptors REV-ERBα and REV-ERBβ which compete at the retinoic acid-related orphan receptor (ROR)– binding elements with the activators RORα, RORβ, and RORγ (22, 23). REV-ERBs and RORs may act to connect the core circadian oscillator to the regulation of clock-controlled genes (CCGs), which in turn regulate metabolism, development and immunity.

At the cellular level, a metabolic oscillator highly conserved through evolution [review in (24)] drives rhythms in oxidation-reduction cycles of peroxiredoxins, reactive oxygen species (ROS) levels and lipid content, which work even in the absence of transcription and interact with the circadian molecular clock (25, 26). Overall, we may infer that the cellular clock is composed of both the molecular clock (transcription–translation-based feedback loop) and the metabolic/redox oscillator [reviewed in (27)].

We have previously reported that *de novo* synthesis of GPLs in different mammalian and nonmammalian vertebrate cells is controlled by a circadian clock, as observed in chicken retinal neurons *in vivo* or *in vitro* (28, 29, 30), in the murine liver (31), as well as in quiescent murine fibroblasts or proliferating glioma cells after synchronization (26, 32) [for review see (27)]. In addition, *ChoKα* expression and/or activity are subject to circadian control in both immortalized cell cultures and mice liver after synchronization (31, 32, 33). In fact, the hepatic circadian clock regulates the *ChoKα* gene expression through the BMAL1-REV-ERBα axis (14, 26). In the liver, the rhythmic regulation of lipid biosynthesis is known to occur (31, 33); however, little is known about the temporal regulation of cellular metabolism in hepatic tumor cells and even less about when the disruption of the intrinsic clock takes place to further alter metabolic pathways.

Here we investigated the rhythmic expression of CGs and CCGs with a special focus on lipid synthesizing enzyme genes, GPL and TG levels, and LD content and formation in dexamethasone (DEX)-synchronized HepG2 control cells [*Bmal1*-wildtype (B-WT)] and *Bmal1*-disrupted (B-D) cells in which the molecular clock is impaired.

## Results

### Temporal regulation of CG expression in HepG2 cells

**Figure 1:**
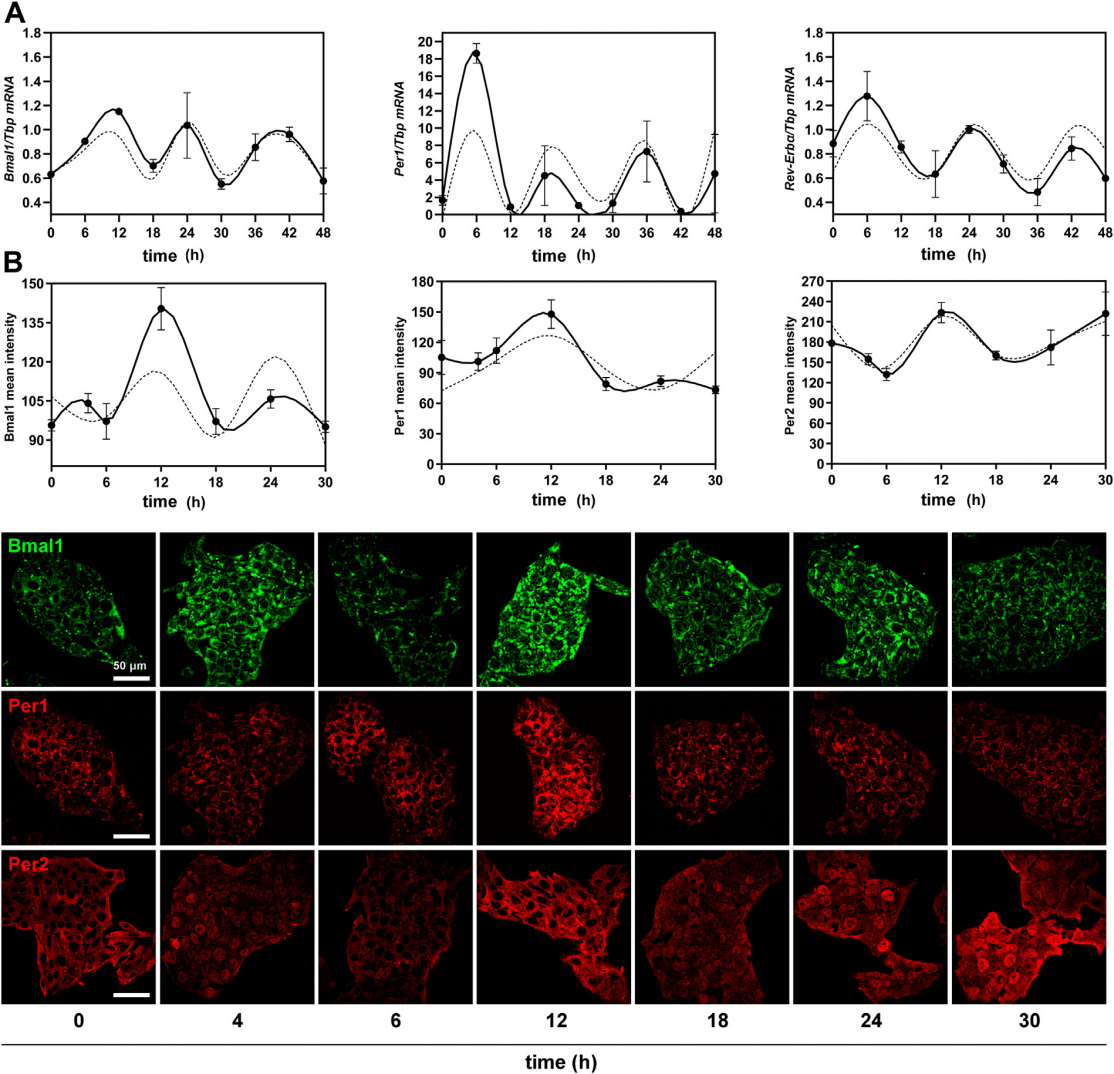
**Circadian characterization of the HepG2 cell cultures for clock gene expression and their periodic analysis.** Cultures of HepG2 cells were synchronized by a 1 h pulse of DEX (100 mM) and then collected at different times for determination of clock gene mRNAs and proteins. *A*, levels of *Bmal1*, *Per1*, and *Rev-Erbα* mRNAs display sustained oscillations with shorter periods than 24 h. Transcript levels were determined by qPCR with *Tbp* as housekeeping gene. Relative quantification is shown in *full lines* and was done by the Pfaffl method using time 24 as calibrator (arbitrarily defined as 1). The periodic adjustment is shown as *dashed lines* (see Table 1 for further information). Significant differences were observed in *Per1* and *Rev-Erbα* (*p* = 0.006 and *p* = 0.02 by ANOVA with Bonferroni test, respectively). See Experimental procedures for further details. *B*, protein levels of BMAL1, PER1, and PER2 assessed by ICC showed significant differences across time (*p* < 0.0001 and *p* = 0.0001 by ANOVA with Bonferroni test, and *p* = 0.01 by ANOVA with K-W test respectively). The periodic analysis is shown in Table1. Scale bar = 50 ⎧m. DEX, dexamethasone; ICC, immunocytochemistry; K-W, Kruskal–Wallis.

**Suppl. Fig. 1:**
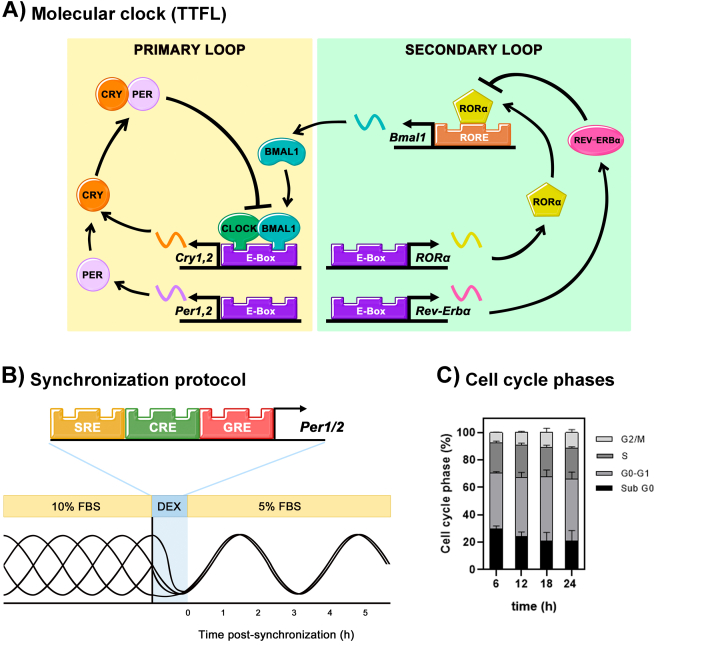


**Suppl. Fig. 2:**
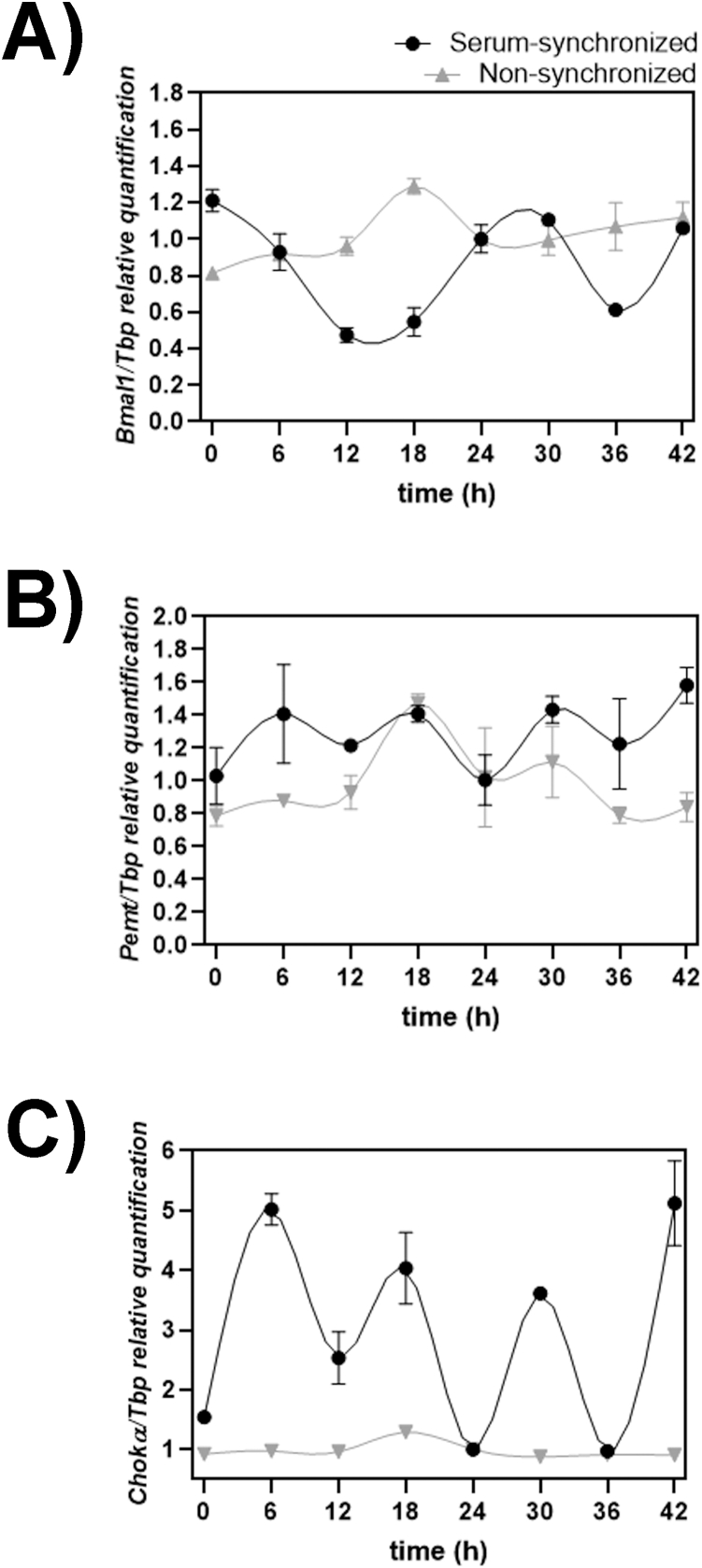


We first investigated the oscillatory capacity of proliferating HepG2 cells synchronized by a DEX shock (time 0) for 48 h (according to protocol illustrated in Fig. S1*B*) by assessing the expression of CGs *Bmal1*, *Per1/2*, and *Rev-Erbα*. Results shown in Figure 1*A* demonstrate that synchronized HepG2 cells displayed a significant temporal variation in mRNA levels for the CGs *Bmal1*, *Per1*, and *Rev-Erbα* (full lines), along the 48 h examined, with markedly different profiles of expression, amplitude, and periodicity (Fig. 1*A* and Table 1). The periods of the oscillations estimated by ARSER analysis (dashed lines) were of ∼14.9, 14.3, and 18.5 h for *Bmal1*, *Per1*, and *Rev-Erbα*, respectively (Table 1). It is noteworthy that *Bmal1* was highly expressed at times 12, 24, and 36 to 42 h after synchronization with lowest levels at 0 to 6, 18, 30, and 48 h, whereas *Per1* and *Rev-Erbα* transcripts exhibited different patterns of expression 6 h advance respect to *Bmal1* mRNA (Fig. 1*A*). In fact, highest levels of mRNA for *Per1* were observed around 6, 18, and 36 h after synchronization, whereas for *Rev-Erbα*, they were at 6, 24, and 42 h postsynchronization. As shown in Table 1, the phases for the oscillations observed were at 9.5, 6, and 6.5 h for *Bmal1*, *Per1*, and *Rev-Erbα*, respectively. Also, a significant oscillation in *Bmal1* mRNA expression was observed in horse serum–synchronized cells with a period of 24 h estimated by RAIN analyses (Fig. S2*A* and Tables S3–S4). In nonsynchronized cells that only received a medium exchange with fresh 5% fetal bovine serum (FBS)–Dulbecco-modified Eagles medium (DMEM) at time 0, an oscillation with substantially diminished amplitude was observed with a period of 24 h by RAIN analysis (Fig. S2*A* and Tables S3–S4).

**Table 1:** Periodic analysis of clock and clock-controlled gene expression in synchronize HepG2 cells

Gene (transcript or protein)	MetaCycle analysis								RAIN analysis	
	Per	Amp	Phase	r2	*p*-value	A.Ph (h)	Min value/time (h)	Max value/time (h)	Period	*p*-value
*Bmal1/Tbp*	14.859	0.282	9.563	0.80	0.0033	12	0.5523/30	1.1511/12	−	N.S.
*Per1/Tbp*	14.257	5.644	5.980	0.75	0.0428	6	0.3645/42	18.6309/6	−	N.S.
*Rev-Erbα/Tbp*	18.539	0.258	6.547	0.96	3.44E-5	6	0.4845/36	1.2765/6	18	0.012
*Pcyt2/Tbp*	15.083	0.241	8.504	0.60	0.0260	6	0.3273/48	1.1478/6	18	0.025
*ChoKα/Tbp*	17.982	0.392	17.951	0.63	0.0218	0	0.5763/6	1.4943/0	18	0.00021
BMAL1	13.248	17.249	10.122	1.00	0.0003	12	95.0946/30	140.3685/12	N.D.	
PER1	22.855	27.378	11.416	1.00	0.0037	12	73.2808/30	147.7562/12	N.D.	

MetaCycle and RAIN analysis was done in R software. For Metacycle analysis, ARSER method was carried out using mean equidistant data. *p* < 0.05 indicates a significant adjustment of the experimental data to the theoretical curve. Transcripts are represented in italics, proteins in capital letters. Per: period, Amp: amplitude, A.Ph.: acrophase, N.D.: non-determined, N.S.: non-significant.

In addition, DEX-synchronized proliferating cells displayed a significant daily fluctuation in levels of BMAL1- PER1- and PER2-like proteins assessed by immunocytochemistry (ICC) (Fig. 1*B*), with highest levels for all proteins examined around 10 to 12 h after synchronization (phases) (Fig. 1*B*, middle and bottom panels, full lines and Table 1). Periods estimated by ARSER analysis were around 13, 23, and 15 h for BMAL1, PER1, and PER2, respectively (Fig. 1*B* dashed lines and Table 1).

### Temporal regulation of phospholipid metabolism in HepG2 cells

**Figure 2:**
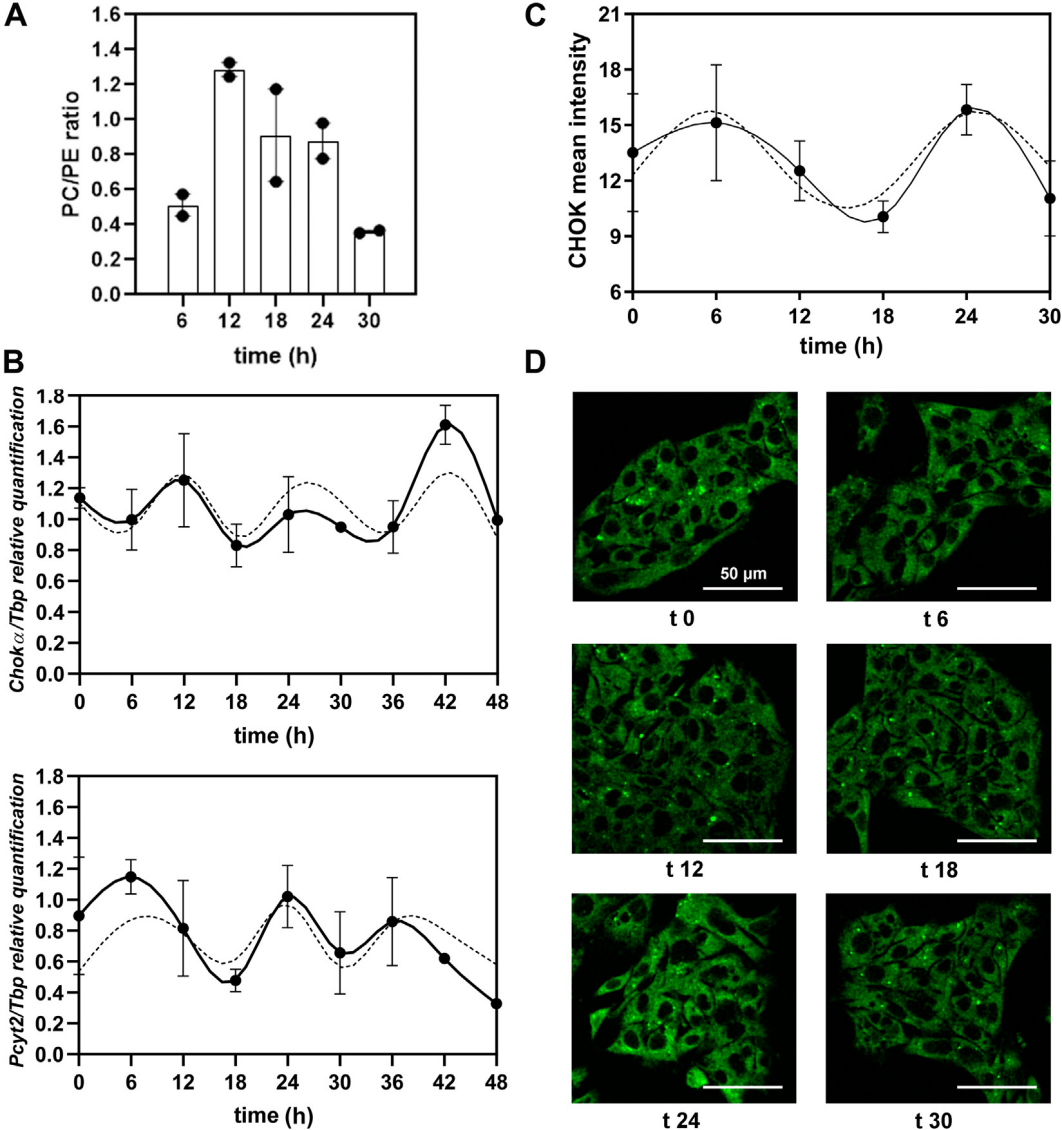
**Temporal regulation of glycerophospholipid (GPL) metabolism in synchronized HepG2 cells.** Cultures of HepG2 cells were synchronized by a 1 h pulse of DEX (100 mM) and then collected at different times. The content of the most abundant GPLs, PC, and PE, the PC/PE ratio and expression of the two key regulatory enzymes for PC and PE biosynthesis, CHOK, and *Pcyt2*, respectively, were assessed across time post synchronization. *A*, the PC/PE ratio displayed significant changes over time in proliferating HepG2 cells after synchronization (*p* = 0.02 by ANOVA with Bonferroni test). *B*, *ChoKα* and *Pcyt2* mRNA levels assessed by qPCR showed significant level differences over time (p≤ 0.05 by ANOVA) while the periodic analysis indicated an oscillation for both messengers with shorter periods than 24 h (see Table 1 for further information). *C*, CHOK protein immunoreactivity evaluated by ICC displayed no significant differences over time (*p* = 0.2 by ANOVA with K-W test) but show highest levels at 24 h after synchronization (*top panel*). *D*, microphotographs show CHOK-like protein immunofluorescence (*green*) denoting a cytoplasmic localization (*bottom panels*) with highest levels at 24 h after synchronization. Scale bar = 50 μm. CHOK, choline kinase; DEX, dexamethasone; ICC, immunocytochemistry; K-W, Kruskal–Wallis; PC, phosphatidylcholine; PE, phosphatidylethanolamine.

In order to investigate the temporal control of GPL synthesis in synchronized HepG2 cells maintained under proliferation, we examined the endogenous levels of PC and PE, the most abundant GPLs and expression for two key regulatory GPL-synthesizing enzymes: *ChoKα* and *Pcyt2* involved in PC and PE biosynthesis, respectively (Fig. 2). Results demonstrated a significant variation across time in percentage levels of PC and PE, respectively (*p* = 0.02 by ANOVA with Bonferroni test, Fig. 2*A*). After considering the sum of both most abundant GPLs as 100%, endogenous levels of PC fluctuated between 55 and 75% across time with the highest levels around 12 to 18 h after synchronization while for PE, values oscillated between 25 and 45% over time. Pairwise comparisons revealed that PC levels at 6 h postsynchronization were significantly lower than those at the peak while for PE, levels at 12 h differed from those at the peak. Moreover, the PC/PE ratio exhibited also a significant temporal variation along the 24 to 30 h (Fig. 2*A*) with the highest levels at 12 h and lowest values at 30 h postsynchronization (*p* = 0.02 by ANOVA with Bonferroni test).

Results also showed that both transcripts examined (*ChoKα* and *Pcyt2*) for the regulatory GPL-synthesizing enzymes exhibited detectable differences in mRNA levels over time (*p*≤ 0.05 by ANOVA) and displayed a significant oscillation along 48 h (Fig. 2*B*, full lines) with periods close to 15 h for *Pcyt2* and 17.9 h for *ChoKα*, and amplitude near 40% (Fig. 2*B*, dashed lines, Table 1) estimated by ARSER analysis.

Moreover, immunoreactivity associated to ChoK*α*-like protein exhibited an apparent but not significant daily fluctuation (*p* = 0.2 by ANOVA with Kruskal–Wallis [K-W]) (Fig. 2*C*) with the highest levels around 6-12 h after synchronization and a period near 19 h (Table 1).

In addition, levels of *Pemt* and *ChoKα* mRNAs displayed a significant oscillation in serum-synchronized cultures (full lines) with a period of 12 h (*p* = 0.0009 and *p* = 0.035 by RAIN analysis, respectively) (Fig. S2, *B* and *C*). Nonsynchronized cells (dashed lines) exhibited a significant rhythmicity for *Pemt* mRNA expression (B, *p* = 0.02 by RAIN analysis) with low amplitude, whereas no time-related differences were seen in *ChoKα* levels along the 42 h examined (Fig. S2 and Tables S3–S4).

Observations demonstrated a significant temporal variation in content of most abundant GPLs (PC and PE), PC/PE ratio, and expression of key GPL-synthesizing enzymes (*ChoKα* and *Pcyt2)* in HepG2 cells, evidencing overall, a time-related control of lipid metabolism within the cell that will be further investigated in following sections.

### Effect of the molecular clock disruption on lipid metabolism of HepG2 cells

**Suppl. Fig. 3:**
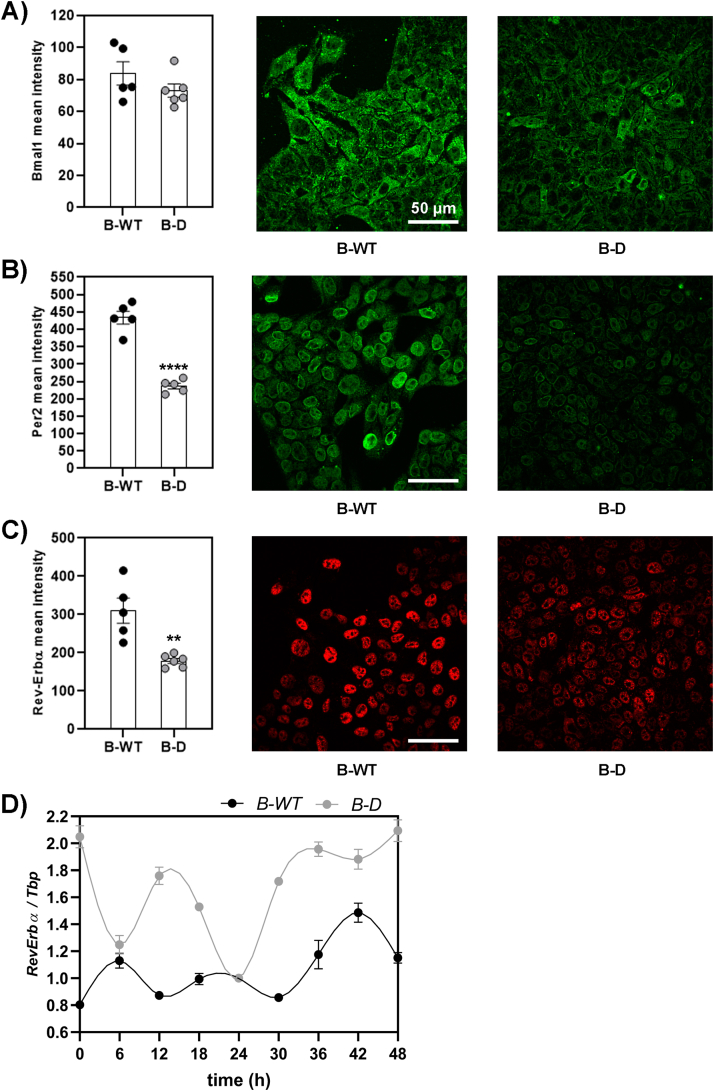


In another series of experiments, we proceeded to disrupt the intrinsic molecular clock in proliferating HepG2 cells by knocking down *Bmal1* expression by CRISPR/Cas9 genomic editing tool (Fig. S3). The disruption of *Bmal1* gene expression was checked by Western blot (WB) and ICC proving a tight reduction, although not significant, in levels of BMAL1 protein in B-D cells (gray bars) as compared with WT controls (B-WT, black bars) (Fig. S3*A*). It is noteworthy that transcript levels of *Rev-Erbα* were significantly increased in B-D cells (Fig. S3*D*, right panel, gray lines) as compared with B-WT cells (black lines). Rhythms observed in B-D cells exhibited a period near 17 h and were remarkably higher in amplitude and a totally different phase (peak at 15 h) as compared with B-WT controls peaking at 4 h postsynchronization (Table 2A). At the protein level, the clock controlled genes PER2 and REV-ERBα exhibited a significant decrease (Fig. S3, *B* and *C*, *p* < 0.0001 and *p* = 0.002, by *t*-test respectively) in B-D cells compared to B-WT cells (controls).

**Table 2:** Periodic analysis of clock-controlled and lipid enzyme gene expression and metabolic parameters in synchronized HepG2 cells wildtype (B-WT) and *Bmal1*-disrupted (B-D) cells

A) Gene (*transcript* or protein)	A. MetaCycle analysis								B. RAIN analysis	
	Per	Amp	Phase	r^2^	*p*-value	A.Ph(h)	MIN value/time (h)	MAX value/time (h)	Period	*p*-value
*Rev-Erbα/Tbp* (B-WT)	18.654	0.173	3.926	0.94	0.0026	42	0.8036/0	1.4862/42	−	N.S.
*Rev-Erbα/Tbp* (B-D)	17.011	0.334	15.207	0.82	0.0216	48	1.0002/24	2.0937/48	−	N.S.
*Pemt/Tbp* (B-WT)	14.499	0.168	3.556	0.86	0.0150	18	0.9571/48	1.5231/18	18	0.012
*Pemt/Tbp* (B-D)	17.509	0.157	14.630	0.95	0.0018	12	0.8399/42	1.3411/12	18	0.003
*ChoKα/Tbp* (B-WT)	17.982	0.392	17.951	0.63	0.0218	0	0.5763/6	1.4943/0	18	0.0002
ChoKα/Tbp (B-D)	14.429	0.012	3.982	0.60	0.1058	6	0.0970/42	0.1419/6	−	N.S.
*Lipin1/Tbp* (B-WT)	16.361	0.002	2.111	0.57	0.1168	0	0.0043/42	0.0134/0	−	N.S.
*Lipin1/Tbp* (B-D)	16.5414	0.024	15.939	0.96	0.0013	0	0.0118/42	0.0754/0	18	0.004
CHOK (B-WT)	19.254	2.742	5.622	0.72	0.0682	24	10.0511/18	15.8280/24	N.D.	
CHOK (B-D)	24.000	1.086	22.973	0.90	0.0143	0	2.4416/30	6.2195/0	N.D.	

**B) LD shape, TG or lactate content**	**Per.**	**Amp.**	**Phase**	**r**^**2**^	***p*****-value**	**A.Ph (h)**	**MIN value/time (h)**		**MAX value/time (h)**	

LDs number (B-WT)	14.291	17.169	6.095	0.99	0.0007	6	37.0000/30		71.8889/6	
LDs number (B-D)	15.393	13.048	7.274	0.92	0.0114	24	24.7857/0		86.3077/24	
LDs % area (B-WT)	14.499	2.768	4.226	0.91	0.0116	18	6.0831/12		12.4846/18	
LDs % area (B-D)	28.514	0.911	16.827	0.80	0.0407	18	1.3664/0		3.7495/18	
LDs average size (B-WT)	25.700	0.213	24.830	0.56	0.1330	0	0.5357/12		1.1797/0	
LDs average size (B-D)	23.482	0.078	16.846	0.97	0.0028	18	0.2509/30		0.4513/18	
Triglyceride (B-WT)	37.192	21.866	33.378	0.59	0.0292	30	24.9948/12		104.7712/36	
Triglyceride (B-D)	34.023	11.691	30.598	0.97	0.0007	36	14.9815/18		45.9808/30	
Lactate (B-WT)	42.468	35.902	26.107	0.69	0.0121	36	37.4149/0		135.9823/36	
Lactate (B-D)	36.963	36.714	32.203	0.37	0.1039	36	56.5148/0		256.0268/36	

Table 2. A). Periodic analysis for transcripts (*italic* letters) or proteins (CAPITAL letters) of clock genes and enzymes involved in the glycerophospholipid biosynthesis are shown in A. MetaCycle analysis using the ARSER method and RAIN analyses were performed for periodic analysis and rhythmic parameters. B) Lipid droplet (LD) number, % area or average size, and contents of triglyceride (TG) and lactate were evaluated over time, and their periodic analysis is shown in B. p < 0.05 indicates a significant effect for the fit of the experimental data with respect to the theoretical curve. See methods for further detail. Per: period, Amp: amplitude, A.Ph: acrophase, N.D.: nondetermined, N.S.: nonsignificant.

**Figure 3:**
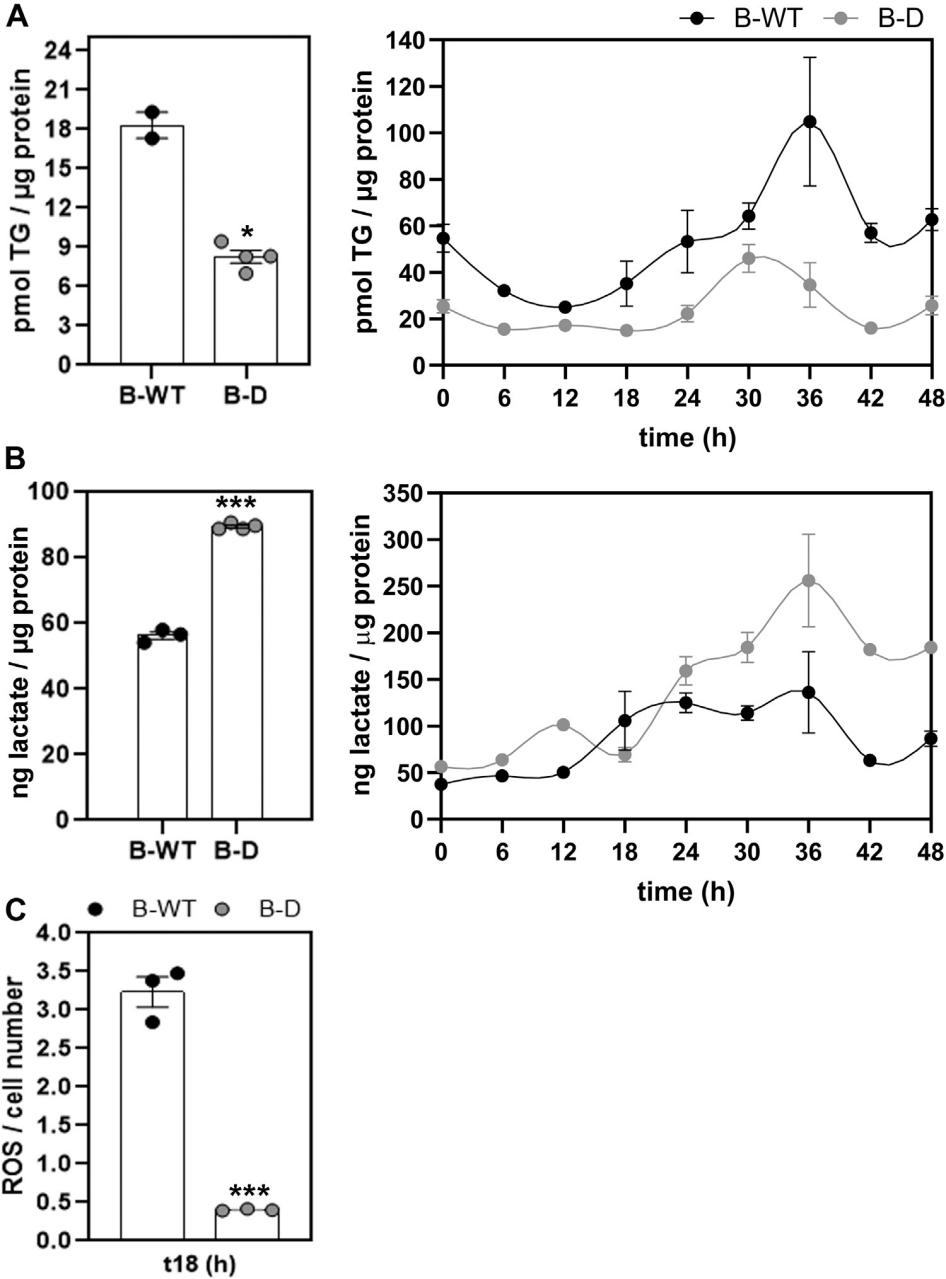
**Metabolic changes in *Bmal1*-disrupted (B-D) cells.** Cultures of B-D or B-WT cells as control were synchronized by a 1 h pulse of DEX (100 mM) and then collected at different times for further determinations. *A*, triglyceride (TG) content was significantly reduced in nonsynchronized B-D cells compared to normal controls (B-WT cells) (*left panel*, *p* = 0.03 by *t* test). Also, TG levels presented significant changes over time postsynchronization in B-WT (*p* = 0.008 by ANOVA with K-W test) as well as in B-D (*p* = 0.007 by ANOVA with K-W test) cells with periods longer than 24 h (*right panel*) (see Table 2B for further detail). *B*, B-D cells exhibited elevated lactate content as compared to B-WT cells (*p* = 0.0002 by *t* test) (*left panel*). In both cell populations, significant changes in lactate levels over time were observed (B-WT *p* = 0.001 and B-D *p* = 0.0004 by ANOVA with K-W test) (*right panel*) fitted to a periodic function with periods longer than 24 h (see Table 2B). *C*, ROS levels were significantly lower in B-D cells than in B-WT cells at 18 h postsynchronization (t 18, *p* = 0.0001 by *t* test). K-W: Kruskal Wallis. ∗*p* < 0.05; ∗∗*p* < 0.01; ∗∗∗*p* < 0.001. B-WT, *Bmal1*-wildtype; DEX, dexamethasone; ROS, reactive oxygen species.

We also observed marked downstream effects presented in Figure 3 on lipid metabolism, lactate, and ROS levels. When endogenous TG levels were determined in proliferating B-WT and B-D cells postsynchronization, we found a substantial decrease in the content of TGs in B-D cells as compared with B-WT controls at all times tested (*p* = 0.03 by *t* test) (Fig. 3*A*, left panel). However, a significant temporal variation in TG levels along 48 h was seen in both cell populations with distinct profiles, amplitudes, and periods far from the circadian scale (Fig. 3*A*, right panel, Table 2B). In B-WT cells, TG levels exhibited a temporal fluctuation with a longer period near 37 h and a peak around 25 h after synchronization (full line) (*p* < 0.008 by K-W). In B-D cells, a temporal variation was observed with a significant lower amplitude and a period of 34 h peaking at 30 h (gray line) (*p* < 0.007 by K-W) (see Table 2B). Furthermore, other strong metabolic effects were found after *Bmal1*-disruption such as a significant increase in lactate levels (*p* = 0.0002 by *t* test) (Fig. 3*B*), mainly at times longer than 24 h postsynchronization, and lower ROS levels at 18 h as compared with B-WT cells (Fig. 3*C*, gray *versus* black bars, *p* = 0.0001 by *t* test).

### Temporal control of lipid metabolism in B-WT and B-D HepG2 cells

**Figure 4:**
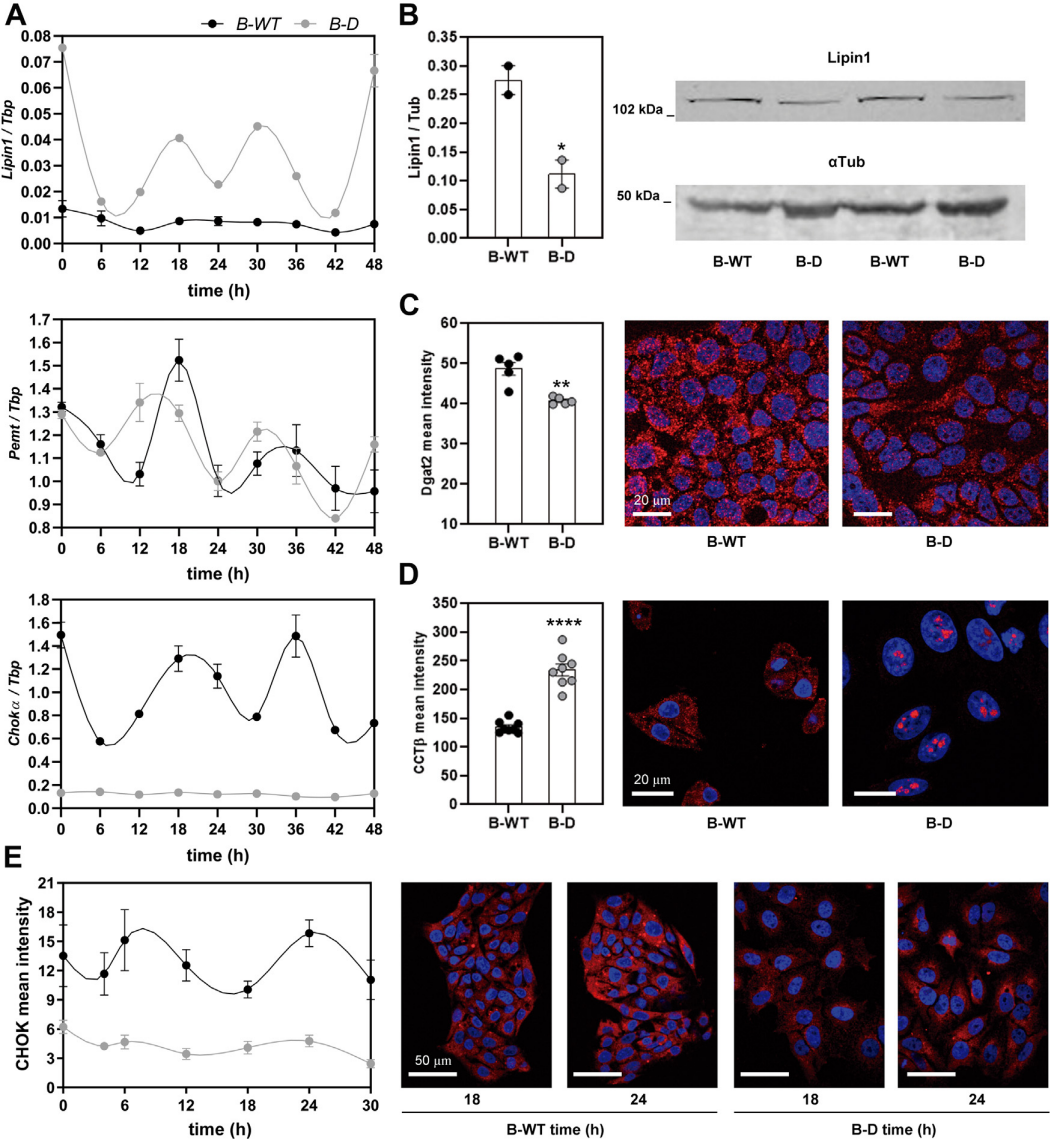
**The expression of glycerophospholipid (GPL) synthesizing enzymes is severely altered in B-D cells.** Cultures of B-D or B-WT cells as control were synchronized by a 1 h pulse of DEX (100 mM) and then collected at different times for mRNA or protein determinations. *A*, mRNA levels for key regulatory enzymes in GPL synthesis were measured by qPCR. Relative levels for *Lipin1* and *Pemt* mRNAs showed significant temporal variation in B-D cells (*gray lines*, *p*= 0.03 by ANOVA with K-W and *p* = 0.0005 by ANOVA with Bonferroni test, respectively) while in B-WT cells no significant differences were observed for *Lipin1* across time; *Pemt* transcripts exhibited a marked phase shift between the two cell populations (see Table 2A). *Lipin1* transcript was overexpressed in B-D cells compared to control (B-WT). *ChoKα* levels showed significant oscillations at postsynchronization times tested in control cells (B-WT, *black line*, *p* = 0.04 by ANOVA with K-W test), but these diminished and lost their rhythmicity when *Bmal1* was disrupted (B-D, *gray line*, *p* = 0.09 by ANOVA with K-W test, ns). *B*, at the protein level, LIPIN1 was assessed by WB and showed a significant reduction in B-D model (*p* = 0.04 by *t* test). *C*, DGAT2 protein was dampened after disruption of *Bmal1* (ICC, *p* = 0.001 by *t* test). *D*, immunoreactivity associated with CCTβ-like protein exhibited a significant accumulation in B-D cells (*p* < 0.0001 by *t* test) together with a notable nucleolar translocation (*red spots* observed in the nucleus stained with DAPI) as compared with its fluorescence levels and cellular location in B-WT cells. *E*, immunofluorescence for CHOKα-like protein displayed significant temporal variations in B-D cells after synchronization (*gray line*, *p* = 0.003 by ANOVA with Bonferroni test) with lower amplitude than control cells (B-W); however, no significant changes were observed in protein levels of B-WT cells (*black line*, *p* = 0.2 by ANOVA with K-W test, ns). As seen for the mRNAs, at the protein level, ChoKα presented a significant decrease after *Bmal1* disruption. Ns, nonsignificant. K-W, Kruskal Wallis. See Table 2A for further detail. ∗*p* < 0.05; ∗∗*p* < 0.01; ∗∗∗∗*p* < 0.0001. B-WT, *Bmal1*-wildtype; BD, *Bmal1*-disrupted; DEX, dexamethasone; ICC, immunocytochemistry.

In order to delve further into lipid metabolism in HCC-derived cells having their intrinsic molecular clock impaired or not, we evaluated lipid biosynthesis by analyzing the expression of diverse key GPL synthesizing enzymes at the mRNA or protein level, intracellular localization, and potential rhythmicity (Fig. 4). *De novo* lipid biosynthesis was assessed by measuring mRNA levels for the key GPL synthesizing enzymes *Lipin1*, *Pemt*, and *ChoKα* along 48 h postsynchronization (Fig. 4*A*). At the protein level, we evaluated the relative levels of LIPIN1 (Fig. 4*B*), CHOKα1/2-like protein (Fig. 4*E*) and the intracellular localization of glycerol-3-phosphate acyltransferase 2 (DGAT2) and of CCTβ (Fig. 4, *C* and *D* respectively). Results clearly show that the relative levels of *Lipin1* mRNAs present dampened the amplitude in B-WT cells (black line) (see Table 1), while levels of *ChoKα* mRNAs oscillated over time with periods shorter than 24 h (*p* = 0.04, Fig. 4*A* lower panel, and Table 2A). In B-D cells, levels of *Lipin1* transcripts were significantly higher than in WT cells and varied across time with a remarkably higher amplitude and values (Fig. 4*A*, upper panel), whereas at the protein level, a significant decrease was observed by WB (Fig. 4*B*). In addition, *Pemt* transcripts showed an increase in B-D cells with significant differences across time (Fig. 4*A*, middle panel, *gray lines*) compared to WT controls (black lines). Levels of *ChoKα* mRNA and protein were significantly reduced in B-D cells over time and rhythms substantially dampened in amplitude as compared to B-WT cells (Fig. 4, *A* and *E* and Table 2A). In addition, levels of DGAT2-and CCTβ-like proteins were differentially regulated compared to WT cells, whereas fluorescence immunoreactivity associated with DGAT2 exhibited a slight decrease (Fig. 4*C*) and the CCTβ-like protein showing a significant increase together with a dramatic change in intracellular localization, becoming fully concentrated in the nuclei of B-D cells (Fig. 4*D*).

### Rhythms in LD levels of HepG2 cells and the effect of *Bmal1*-disruption

**Figure 5:**
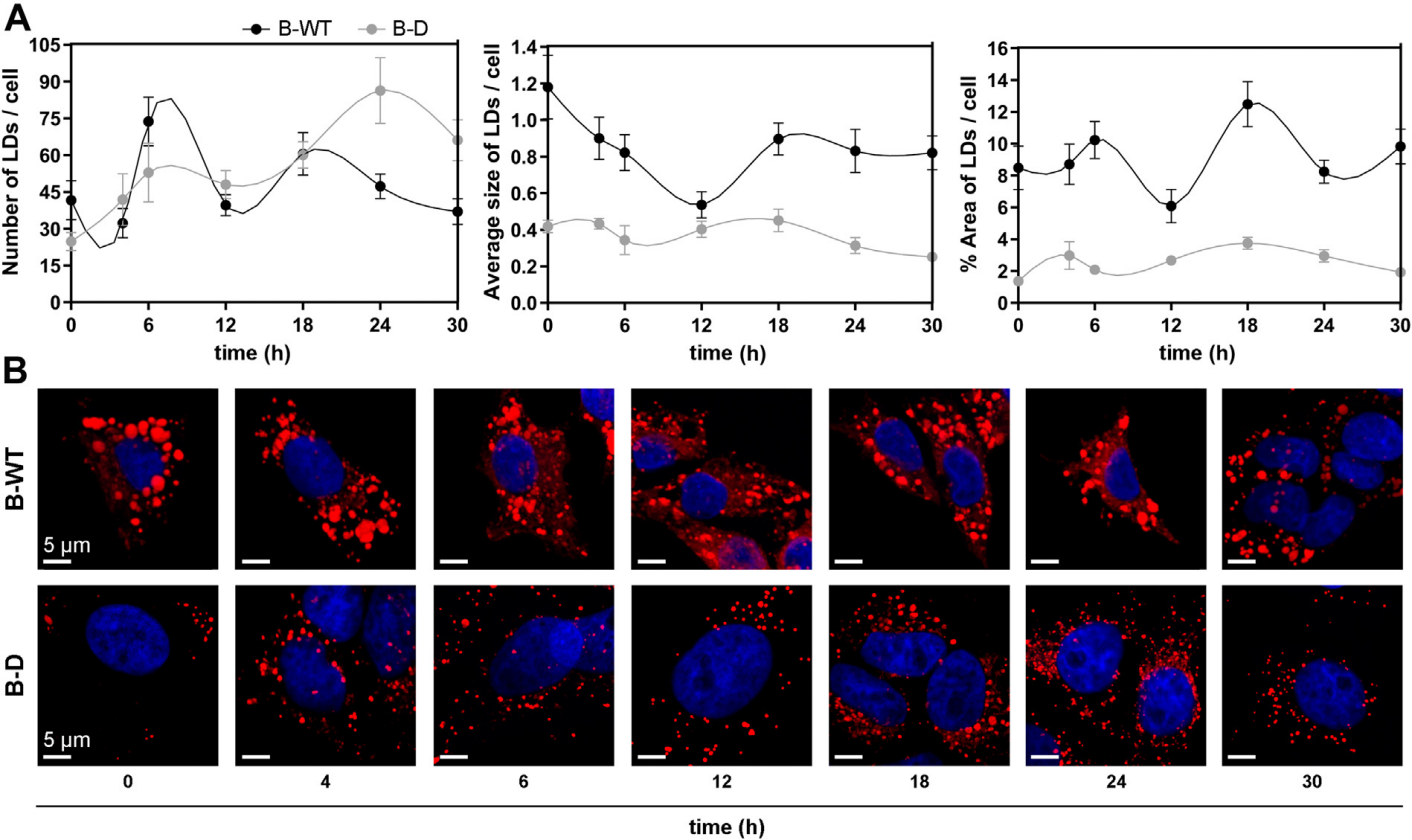
**Effects on lipid droplet (LD) formation (content and size) after *Bmal1* disruption in HepG2 cells.** Cultures of B-D or B-WT cells as control were synchronized by a 1 h pulse of DEX (100 mM) and then collected at different times for LD assessment. *A*, formation of LDs (number, size and area) was evaluated over time postsynchronization in both HepG2 cell groups by Nile Red staining. B-WT cells presented significant temporal changes in number, % area, and average size of LD staining (*p* = 0.001 and *p* = 0.01 by ANOVA with Bonferroni test, and *p* = 0.04 by ANOVA with K-W test, respectively) as well as in B-D cells (number: *p* = 0.0002, size: *p* = 0.0002, % area: *p* = 0.01). After *Bmal1* disruption, minor differences were observed in the number of LDs, but the average size and the % area were notably reduced in B-D cells (approximately a 50% and a 75% respectively) compared to B-WT control cells. *B*, representative microphotographs of LD staining across time. LD fluorescence staining showed in *red*, and nuclei stained with DAPI in *blue*. Scale bar = 5 ⎧m. BD, *Bmal1*-disrupted; B-WT, *Bmal1*-wildtype; DEX, dexamethasone; K-W, Kruskal–Wallis.

In another series of experiments, we evaluated the temporal regulation of lipid accumulation in LDs in both groups of synchronized cells (B-WT and B-D) assessing the number, average size, and percentage area of LDs along 30 h after Nile Red staining (Fig. 5 and Table 2B). First, LDs in WT cells (Fig. 5*A*, black lines) showed significantly higher average size values and % area than in B-D cells at all times tested (Fig. 5*A*, gray lines). Furthermore, B-WT cells exhibited marked oscillations in % area and number of LDs with a period ∼14 h (black lines), whereas for B-D cells, LDs oscillations in size and area were significantly diminished with longer periods. Remarkably, for the number of LDs, oscillations were observed in both conditions but with slightly different phases and amplitudes (Table 2B).

## Discussion

The mammalian liver constitutes a very exciting model of peripheral oscillator to investigate the circadian regulation of cellular metabolisms and to elucidate the link between the molecular clock and hepatic metabolic processes. Moreover, it is now known that the disruption of the molecular circadian clock may cause a number of metabolic disorders, grouped as the metabolic syndrome and involving obesity, type II-diabetes, hyperlipidemia, among others, and higher risk of cancer (17, 34, 35, 36, 37). Our observations reveal for the first time that hepatic tumor HepG2 cells kept under proliferation contain a functional intrinsic cellular clock that tightly controls diverse cellular and metabolic functions, especially those related to GPL biosynthesis, TG production, and LD accumulation. HepG2 cells kept in culture and once synchronized by a pulse of DEX (synthetic glucocorticoid) were able to retain the oscillatory condition under proliferation, making them a potential therapeutic target from a chronobiological point of view as recently shown in gliomas of different origins (26, 38, 39).

Glucocorticoids have been shown to inhibit cell growth, entrain the cell cycle, and induce arrest, potentially leading to apoptosis. Brief pulses of DEX can elicit phase shifts in the liver, kidney, and heart and in cell culture can induce and entrain circadian expression of core CGs *Per1/2* and Cry1 in different cell types (reviewed in (40)). In our experimental model, 1-h pulse of DEX (100 mM) was used as a synchronizing signal to adjust individual cellular oscillators in culture driving both transcriptional and metabolic rhythms (Figure 1, Figure 2, Figure 3, Figure 4, Figure 5). Glucocorticoids can act through glucocorticoid receptor–independent pathways or by the glucocorticoid receptors and glucocorticoid-response elements such as those present in *Per1,2* and in some CCG promoters. Nevertheless, other extracellular signals such as a 2 h-serum shock or to a less extent, fresh medium exchange, were also able to synchronize the circadian clock in HepG2 cells driving sustained rhythms in CGs, CCGs, and lipid synthesizing enzymes genes and both in levels of mRNA and metabolic activity of CYP3A4 (heme-containing monooxigenases) (Fig. S1 of this paper, Table S3) (41). By contrast, under prolonged treatment (1 μM for 16–40 h), DEX exhibits antiinflammatory properties and has a protective influence on hepatocellular lipid metabolism and FA transporter expression and synthesis. In particular, the prolonged treatment of HepG2 cells with DEX enhanced lipid transport to the cell and resulted in increased triacylglycerol, DG, and ceramide accumulation (42).

Notably, the oscillations reported here at the level of mRNA and proteins for CGs, CCGs, and also regulatory enzymes for GPL synthesis displayed bimodal rhythms on the circadian base with average periods ∼16 h (see Tables 1 and 2) as reported in other cellular tumor models (26, 38) [see Guido *et al* 2021 for review (27)]. Although the molecular basis of ultradian rhythms (periods shorter than 20 h) is unknown, it has been proposed that an interplay between two different circadian regulatory components (transcription factors, nutrient availability, extracellular signals, etc.) with opposite phases might induce such rhythms for a given gene or protein (43). This hypothesis further links this rhythmic behavior with a particular metabolism or cellular process and the proliferative state of the cell (arrest or proliferation), particularly for tumor cells. The discrepancies observed between the appearance of mRNAs and their proteins for some CGs and CCGs (Figs. 1 and 2) can be due to rhythms of different periods between transcripts and proteins oscillations and different mechanisms related to the synthesis, degradation, and/or posttranslational modifications as observed particularly in tumor cells (26, 38).

It is noteworthy that GPL metabolism in the mammalian liver is subject to precise temporal control, as demonstrated in animals synchronized to light/dark cycles or maintained in constant darkness (31). Temporal variations were observed in endogenous GPL content and in the activity and expression of key biosynthetic GPL enzymes such as diverse lyso-GPL acyl transferases and LIPIN1.

A number of human studies and their counterpart in animal models strongly show that the PC/PE molar ratio balance is a key determinant of liver health and when abnormal becomes a marker of hepatic diseases. PC synthesis impairment in the liver and changes in the composition of hepatic GPLs have been related to fatty liver disease, liver failure, impaired liver regeneration after surgery, and metabolic disorders (44). Abnormal values (high or low) of cellular PC, PE, or PC/PE molar ratios can also be associated with problems in energy metabolism and have been linked to disease progression. In fact, the PC/PE ratio is a key regulator of cell membrane integrity, playing an important role in the progression of steatosis into steatohepatitis since a significant decrease in the PC/PE ratio leads to loss of membrane integrity and the resulting cell damage promotes an inflammatory response typical of steatohepatitis (44). These observations undoubtedly have clinical implications as patients with nonalcoholic steatohepatitis have a lower PC/PE ratio than normal controls. Moreover, in a number of animal models, PC/PE levels have been implicated in the pathogenesis of several metabolic disorders (9).

In our study involving tumor cells, when the molecular clock was disrupted, a remarkably strong metabolic impairment was observed that significantly affected the whole metabolic status of the cells. Notably, an abnormal increase in lactate levels was found and a substantial dampening of lipid/energetic processes with a severe decrease in endogenous content of TG levels, LD accumulation, and ROS content. Our results clearly demonstrate a significant decrease in the endogenous content of TG in B-D HepG2 cells (Fig. 3) and consequently LD content (Fig. 5) compared to WT cells. These observations could be due to a marked decrease in the expression of key GPL synthesizing enzymes, particularly *ChoK* mRNA and protein (Fig. 4, *A* and *E*, respectively), LIPIN1 protein (Fig. 4*B*), acyltransferases expression, and relocalization of CCTβ to the nuclei. (Fig. 4*D*). Considering that PC is regulated by the circadian clock through the *Bmal1/Rev-Erbα/ChoKα* loop (31, 33), the availability of PC could be an essential regulator of the size and number of LDs. The PC to PE ratio has been shown to regulate cell membrane integrity and to play a key role in avoiding apoptosis (45). Alterations in the PC/PE ratio have also been reported to modulate mitochondrial function and the size and metabolism of LDs (46). Our observations lead us to speculate that a compensatory mechanism attempted to cope with this abnormal situation of metabolic misbalance through higher levels of *Lipin1* and *Rev-Erb*α mRNA expression (Figs. 4*A* and 3*C* right panel, respectively). However, at the protein level, LIPIN1 and REV-ERBα were substantially downregulated in B-D cells, further supporting the general decrease in GPL, TG levels, and PC biosynthesis.

The metabolic misbalance observed in our study after *Bmal1*-disruption in HCC cells is further reinforced by the high levels of lactate detected (Fig. 3*B*). Although lactate is produced by most tissues in the human body under normal conditions, it is rapidly cleared mainly by the liver. Under pathological conditions, lactate elevation may be caused by increased production, decreased clearance, or a combination of both and is mainly due to a hypermetabolic state, macrocirculatory and/or microcirculatory dysfunction (hypoperfusion: reduced amount of blood flow), and mitochondrial dysfunction (including potential lack of key enzymatic cofactors), among others. Liver dysfunction may contribute to both increased production and decreased clearance of lactate (47) giving rise to the accumulation observed in our experiments in B-D cells. An energy switch known as the Warburg effect is present in cancer cells and is characterized by an increase in lactate production. In this condition, the main source of metabolic energy comes from an aerobic glycolysis instead of the usual mitochondrial oxidative phosphorylation. It is hypothesized that the Warburg effect gives tumor cells the ability to acquire and metabolize nutrients in a manner conducive to proliferation rather than efficient ATP production (48). Moreover, lactic acidosis occurs when there is too much lactic acid in the organism as a consequence of a number of pathological processes involving cancer, seizures, liver failure, prolonged lack of oxygen, and low blood sugar.

Lactate is the obligatory product of glycolysis under fully aerobic conditions (49). As a result of molecular clock disruption (B-D cells), HepG2 tumor cells appear to change the direction of their energy metabolism toward glycolysis instead of ATP generation through mitochondrial respiration. This is supported not only by the higher levels of lactate assessed in B-D cells compared to controls but also by the lower levels of ROS—products of mitochondrial oxidative phosphorylation—observed after BMAL1 knockdown (Fig. 3*C*). In B-D cells, it is suggested that a major tumor phenotype is observed, with higher lactate levels potentially underlying an exacerbated glycolytic metabolism when the molecular clock control is relaxed. In this connection, in the human osteosarcoma U2OS cell line, BMAL1 KD diminished metabolite rhythms, while CRY1 or CRY2 perturbation generally shortened/lengthened rhythms, respectively (50). Moreover, BMAL1 KD resulted in loss of rhythmicity in most metabolites while the glycolytic metabolites lactate and bisphosphoglycerate cycled despite not cycling under control (WT) conditions. These observations suggest that cellular metabolic rhythms may largely depend on an intact transcriptional oscillator; nevertheless, as we also found in HepG2 (see Fig. 4 and Table 2) and T98G cells, some independent metabolic oscillators may also exist (26). In addition, *Bmal1* knocked-down glioma cells exhibited a higher tumor growth rate when injected into mice than control glioma cells, reflecting a major tumor phenotype (38).

It is noteworthy that enhancing clock function inhibits tumorgenicity (51, 52) and that clock robustness is further enhanced in human nontumor samples as compared to tumor cells (53). Furthermore, even in specific circumstances when they have lost their rhythmicity in clock and CCG expression by *Bmal1* KD or when the period of oscillation is shortened under proliferation, T98G cells still continue to oscillate for a number of metabolic/redox activities (GPL biosynthesis, redox state cycles, etc.) with a similar period to that under arrest (26).

In conclusion, HepG2 cells maintained in a proliferative condition keep a functional circadian clock that temporally controls cellular metabolism. According to our findings, the temporal patterns observed in lipid metabolism of control tumor cells (B-WT cells) were severely affected and mostly dampened after the disruption of *Bmal1*, a key component of the molecular clock (B-D cells). These findings not only highlight the tight connection between the circadian system and lipid metabolism but also reveal a metabolic susceptibility of HepG2 cancer cells to circadian disturbance, which can be used in chronotherapy to improve therapeutic efficacy. Although further studies will be required in order to elucidate the homeostatic misbalance that take places in B-D cells, based on our results, we can hypothesize that a metabolic switch occurs to ensure cell survival. From an energetic point of view, it would appear that increased lactate levels and decreased ROS content, possibly implying less efficient ATP production, cause a Warburg effect to take place in such a way that B-D cells somehow maintain active cell division. In addition, it can be speculated that a mechanism of autophagy rather than apoptosis has been activated in *Bmal1*-disrupted cells, allowing cells to keep the metabolic rate and flow of metabolites needed to proliferate in the absence of biosynthetic GPL pathways (diminished Kennedy pathway observed in B-D model).

**Figure 6:**
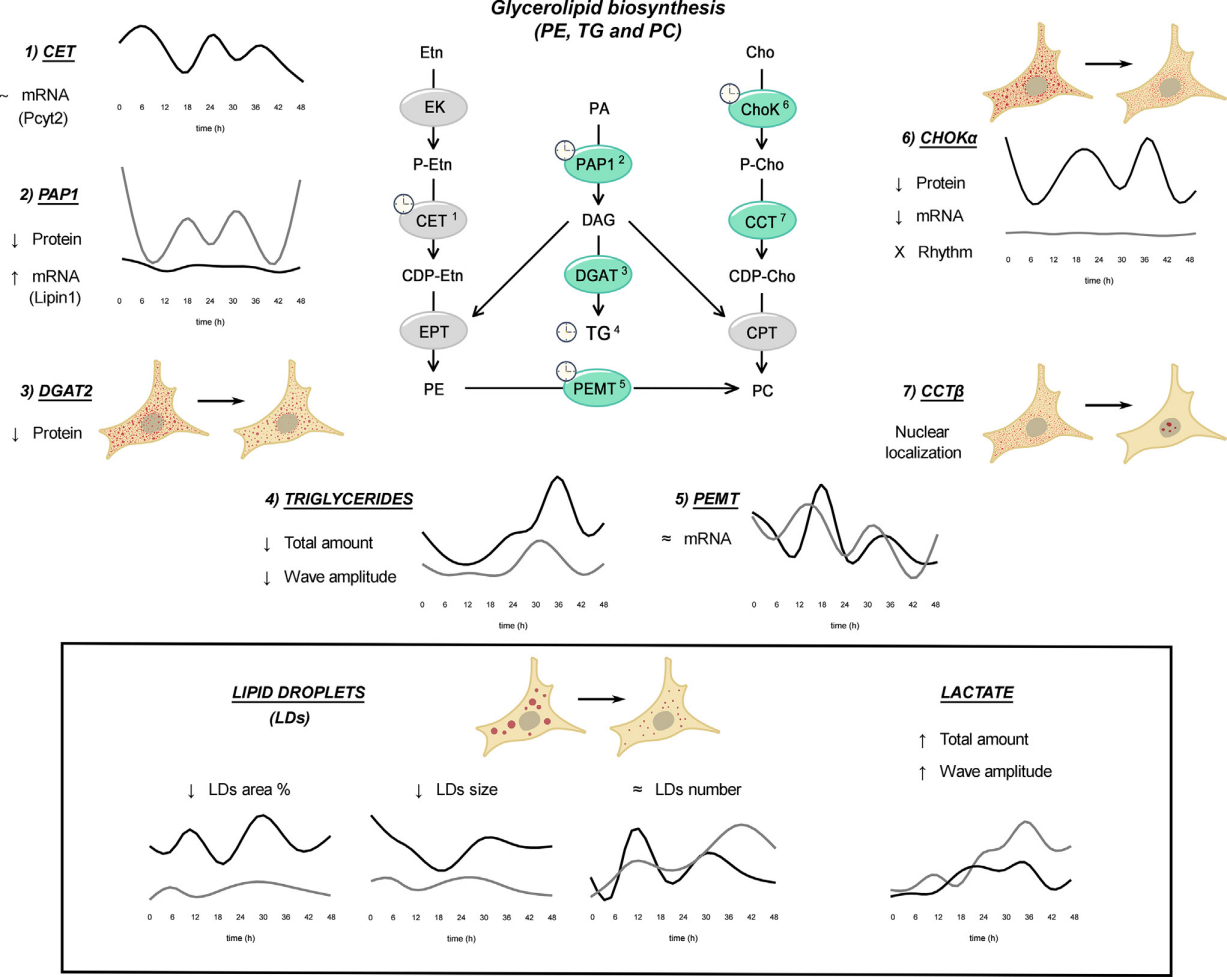
**Temporal regulation of glycerolipid metabolism in HepG2 cells.** The most abundant GPLs in hepatic cells, PC and PE, are mainly synthetized by the *de novo* Kennedy pathway. The enzymes and pathways labeled with a clock indicate a significant temporal variation at the level of mRNAs and/or proteins or endogenous lipid content in cultures of HepG2 cells (*Bmal1*-WT) synchronized by a pulse of DEX at time 0. The endogenous GPL content, the PC/PE ratio, the expression of key regulatory GPL synthesizing enzymes (mRNAs and proteins) (*black line*) such as for *Chokα*, *Pemt*, *Pcyt2*, and *Lipin1* for PC, PE, or TG biosynthesis, together with LD levels (size and number) displayed sustained temporal oscillations with periods ranging from 12 to 24 h. By contrast, when the molecular clock gene *Bmal1* was knocked down (B-D cells) (*gray line*), most transcriptional and metabolic rhythms were severely perturbed, some of them damped with a decreased amplitude (*Chokα*), others upregulated (*Lipin1* mRNA) or phase shifted (*Pemt*). In addition, the rate limiting enzyme for PC synthesis, CCTβ, changed its intracellular location from cytoplasm in B-WT cells to nucleus in B-D cells, while lactate levels increased significantly, and LDs decreased in amplitude for area and size. Overall, the circadian disruption in HepG2 cell cultures strongly suggests a tight link between the molecular circadian clock and GPL metabolism. Cho, choline; ChoK, choline kinase; CCT, CTP:phosphocholine cytidylyltransferase; CDP-Cho, cytidine diphosphate choline; CPT, CDP-choline:diacylglycerol cholinephosphotransferase; DAG, diacylglycerol; DGAT, diacylglycerol acyltransferase, CDP-Etn, cytidine diphosphate ethanolamine; CET, CDP-ethanolamine:diacylglycerol ethanolaminephosphotransferase encoded by *Pcyt2* gene; EPT, CDP-ethanolamine:diacylglycerol ethanolaminephosphotransferase; Etn, ethanolamine; EK, ethanolamine kinase; P-Etn, phospho ethanolamine; G3P, the glycerol-3-phosphate; GPAT, G3P acyltransferase; GPLs, glycerophospholipids; P-Cho, phosphocholine; PC, phosphatidylcholine; PEMT, phosphatidylethanolamine methyl transferase; PS, phosphatidylserine; PE, phosphatidylethanolamine; TAG, triacylglycerol; LD, lipid droplet; phosphatidate phosphohydrolase 1 (PAP1) encoded by LIPIN1.

Overall, it is clear that GPL synthesis and correct circadian regulation are essential for tumor cell survival and thus for hepatic tumor cell proliferation and growth. It is noteworthy that alterations in the level and proportion of GPLs and TGs trigger metabolic disorders like fatty liver, obesity, and insulin resistance and have important cellular implications as shown in our HCC cell model after disruption of the molecular circadian clock (see Fig. 6). These circadian alterations may severely perturb the entire tumor cell metabolism, making these cells a novel target for chronotherapeutic liver treatment and for further metabolic studies.

## Experimental procedures

### Cell cultures

HepG2 cells (ATCC Cat# HB-8065, RRID: CVCL0027) are derived from the human HCC. HepG2 cells tested negative for *mycoplasma* contamination. Cells were grown in DMEM (Gibco, BRL, Invitrogen) supplemented with 10% FBS (Gibco or Natocor), according to (27). After the desired degree of confluence had been achieved in a CO_2_ incubator at 37 °C for 24 to 48 h, cells were synchronized by a 1-h shock with 100 nM DEX (Sigma, D4902) in serum-free medium, exhaustively washed with PBS 1× and allowed to grow in the presence of 5% FBS-DMEM. After DEX synchronization (time 0), cells were collected at 4 to 6 h intervals during 30 to 48 h (Fig. S1*B*).

The solution of 100 nM DEX was prepared from a stock of 10 mM DEX dissolved in ethanol. From this stock, we prepared a 1 mM working solution in PBS, and then aliquots were resuspended in DMEM to a final concentration of 100 nM.

### Horse serum synchronization

In another series of experiments, HepG2 cell cultures were synchronized by a 2-h shock with 50% horse serum (Gibco) medium and allowed to grow in the presence of 5% FBS-DMEM. After horse serum synchronization (time 0), cells were washed with 1× PBS and collected at 6-h intervals during 48 h (Fig. S2). Nonsynchronized cells maintained with 5% FBS-DMEM were subject to fresh medium exchange at time 0, and then collected at 6-h intervals for 48 h as control samples.

### Cell cycle progression analysis in HepG2 cells

HepG2 cells were synchronized as previously described, and samples were collected at 6 h intervals during 24 h (Fig. S1, *B* and *C*). Cells were harvested by trypsinization at different times post synchronization, washed in cold PBS, and fixed with cold 70% ethanol at 20 °C for at least 24 h. Cell pellets were resuspended in 150 μl of staining solution (PBS containing 50 μg/ml propidium iodide and 10 μg RNAse A) as reported (27, 32). Cell cycle analysis was performed with 50,000 cells on a flow cytometer (DB Bioscience). The analysis program used was ModFit 5.0 software (Verity Software House).

### Redox state in HepG2 cells

The redox state was analyzed in B-WT and B-D HepG2 cells. Briefly, the culture medium was removed, and cells were washed with cold PBS 1× and harvested by trypsinization. Next, 4 x 10^5^ cells were incubated with 2,7-dichlorodihydrofluorescein diacetate at 2 μM final concentration for 40 min at 37 °C, washed twice with PBS 1×; the fluorescence intensity of 50,000 events was then measured by flow cytometry at 530 nm when the sample was excited at 485 nm (27, 54). 2,7-dichlorodihydrofluorescein diacetate is a fluorogenic dye that measures hydroxyl, peroxyl, and other ROS activity within the cell. Positive controls were done with an initial incubation with H_2_O_2_ over 15 min. Cells without the fluorescent indicator were used as negative control, and propidium iodide (50 μg/ml) staining was used to discriminate viable cells. *FlowJo* software was used to analyze fluorescence intensity (Verity Software House).

### Immunocytochemistry

ICC was performed as described (27). Briefly, cultured cells were fixed for 15 min in 4% paraformaldehyde in PBS and 10 min in methanol. Coverslips were washed in PBS, treated with blocking buffer (PBS supplemented with 0.1% BSA, 0.1% Tween 20, and 0.1% glycine), and incubated overnight with primary antibodies (Table S1) at 4 °C. Coverslips were washed three times in PBS 1× buffer and incubated with goat anti-rabbit IgG (Jackson 549 antibody 1:1000) or goat anti-mousse IgG (Jackson 488 antibody 1:1000) for 1 h at room temperature. Coverslips were finally washed thoroughly and visualized by confocal microscopy (FV1200; Olympus). Cellular nuclei were stained with DAPI.

### Western blot

Cells were cultured and synchronized as previously described. Culture medium was removed, and attached cells were washed with cold PBS 1× buffer. Western blot was performed according to Rios MN *et al* 2019 (55). Briefly, a RIPA buffer (50 mM Tris–HCl, pH 8.0, 150 mM sodium chloride, 1.0% Igepal NP-40, 0.5% sodium-deoxycholate, and 0.1% sodium dodecyl sulfate) was added; cells were incubated on ice for 10 min and then collected by scraping and sonicated three times for 5 s. A protease inhibitor (Sigma–Aldrich) was then added, and samples were stored at −20 °C until use. For electrophoresis determination, sample buffer was added and separation was done on a 12% polyacrylamide SDS-gel (75 mg total protein/lane), transferred onto polyvinylidenefluoride membranes, blocked for 1 h at room temperature (RT) with 5% BSA in PBS, and then incubated overnight at 4 °C with specific antibodies (Table S1) in an incubation buffer (3% BSA, 0.1% Tween 20, 1% glycine, 0.02% sodium azide in PBS). Membranes were washed three times for 15 min each in washing buffer (0.1% Tween 20 in PBS) and incubated with the corresponding secondary antibody in the incubation buffer during 1 h RT, followed by three washes with washing buffer for 15 min each. Membranes were scanned using an Odyssey IR Imager (LI-COR Biosciences). Membranes were also incubated in blocking buffer containing α-tubulin antibody.

### RNA isolation and reverse transcription

Total RNA was extracted from cell homogenates using TRIzol (Invitrogen) or Tri Reagent (MRC) reagent following the manufacturer's specifications. The yield and purity of RNA were estimated by absorbance at 260/280 nm. Total RNA (2 μg) utilized as a template for the cDNA synthesis reaction using MMLV reverse transcriptase (Promega) and Oligo-dT (Biodynamics) in a final volume of 25 μl according to the manufacturer's indications.

### Real-time PCR

Quantitative RT-PCR was performed using SYBR Green in a Rotor Q Gene (Qiagen) or a CFX96 Touch Real-Time PCR Detection System (Bio-Rad). The primer/probe sequences are summarized in Table S2. The amplification mix contained 1.5 μl of the cDNA, 0.45 μl of forward-reverse 10 μM primers, and 7.5 μl of Master Mix 2× (Biodynamics) in a total volume of 15 μl. The cycling conditions were 5 min at 95 °C, and 40 cycles of 95 °C for 30 s, 60 °C or 55 °C for 30, and 72 ºC for 30 s. Melt curves were done from 55 °C to 97 °C, with an increment of 1 °C. The standard curve linearity and PCR efficiency (E) were optimized. We used the Pfaffl method for quantification (56), and TBP was used as reference gene for normalization (32). For circadian analysis, the time “24 h” was used as calibrator and arbitrarily set as value 1. Each RT-PCR quantification was performed at least in duplicate for each sample (n = 2–4/sample) from two independent experiments.

### Disruption of *Bmal1* expression in HepG2 cells by CRISPR/Cas9

*Bmal1* expression was disrupted in transfected HepG2 cells using the CRISPR/Cas9 genomic editing tool as previously described (38). Briefly, we designed single guide RNAs specifically targeting the 5′UTR region of the human *Bmal1* gene, which we subcloned into the PX459 vector (Addgene) to obtain the PX459-*Bmal1* plasmid. The primer sequence corresponding to the single guide RNA was 5′CTGGCTAGAGTGTATACGTT 3′ and the complementary sequence 5′ GACCGATCTCACATATGCAA 3′. HepG2 wildtype (B-WT) cells were transfected with Lipofectamine 2000 (Invitrogen) and selected with puromycin (2 μg/ml) for 24 h. Transfected cells were diluted in a 96-well plate for clone selection. Cells carrying the disruption of *Bmal1* gene expression (B-D) were checked by WB and ICC (Fig. S2*A*) showing that *Bmal1* content was lower in B-D cells than in the WT control cells. Significant effects were further found on the expression of the downstream target genes PER2 and REV-ERBα assessed by ICC (Fig. S2).

### TG levels quantification

HepG2 cells were grown in DMEM supplemented with 10% FBS, synchronized, and collected at different times as previously described. Cells were washed with cold PBS 1×, scraped with 180 μl of fresh PBS 1×, and sonicated 3 times for 5 s. After centrifugation, 25 μl of supernatant was separated for protein determination. From the remaining extract, 70 μl was used for TG determination with 500 μl of TG Color reagent following the manufacturer's specifications (Wiener Lab). A standard curve was performed with glycerol. Samples and standards were incubated 5 min at 37 °C and quantified at 505 nm by spectrofluorometry. TG quantification was normalized by protein content assessed by Bradford's method.

### Lactate assay

HepG2 intracellular content was isolated as described in the previous section for TG assessment. For lactate determination, 12 μl of samples were diluted in a final volume of 500 μl following the manufacturer's specifications (Wiener Lab.). A standard curve was performed with DL-lactate (Sigma). The content of lactate in samples was quantified at 550 nm by spectrofluorometry and normalized by protein content.

### Lipid extraction

HepG2 cells were grown in DMEM supplemented with 10% FBS until confluence. Cells were synchronized as previously described, harvested by trypsinization, and subject to lipid extraction according to Chao HC, *et al* 2017 (57). Briefly, lipids were extracted with 500 μl of methanol:water (4:1), 400 μl of chloroform (two steps of 200 μl each one), and 200 μl of water. After mixing by vortex and centrifugation, 400 μl of the lipid layer was collected, dried under nitrogen stream, and saved at −80 °C until use.

### Chromatographic separation of individual glycerophospholipids

Individual GPLs were separated by TLC using silica gel G plates previously washed with 1:1 chloroform and methanol system and impregnated in 1.2% H_3_BO_3_ (in ethanol:water 1:1, v/v) (from Macherey-Nagel). The solvent system ethyl acetate:chloroform: isopropanol: KCl 0.25%: methanol (25:25:25:9:10) constituted the mobile phase. Hundred microliter per sample were separated and tested with standards of phosphatidic acid, PE, PC phosphatidylserine, and sphingomyelin. The plate was washed in water and hot air-dried, and then PLs were revealed in 3% cupric acetate (in 8% phosphoric acid solution). After 10 min of air-drying, bands were revealed by 10 min incubation at 170 °C. In the latter case, the plate was scanned and the signal intensities from the individual GPLs bands were calculated as the ratio between the signals of one individual GPL of each sample in percent of pixels and the total pixels for each sample using the Gel Pro Analyzer software. The PC/PE ratio was calculated as the ratio between the PC and PE content of each sample irrespective of the form of normalization.

### Determination of LDs

HepG2 cells were grown in coverslips. To stain LDs, culture medium was removed, and cells were fixed for 15 min in 4% paraformaldehyde in PBS and washed twice with PBS 1×. Then, cells were incubated with Nile Red (1 μg/ml, Sigma) for 15 min at room temperature protected from light. Coverslips were washed thoroughly and visualized by confocal microscopy at 60× objective (FV1200; Olympus). A Z stack scan was performed to cover the entire volume of cells, acquiring 20 slides every 0.12 μm. Cellular nuclei were visualized by DAPI staining. Average size, percentage area, and number quantification of LDs were carried out with *ImageJ* software and quantified from the z project of total slides.

## Statistics

Statistical analyses involved a one- or two-way ANOVA to test the time or disruption effects with K-W post hoc test when the normality of residuals was infringed. Pairwise comparisons involved the Student *t* test or Bonferroni when appropriate. Data are expressed as mean ± SEM. In all cases, significance was considered at *p* < 0.05 (see Table S4 for further detail).

### Periodic analysis

For periodic analysis, we performed a MetaCycle analysis using a meta2d function for detecting rhythmic signals in R Studio program (R Core Team 2017, https://www.r-project.org/), which incorporates multiple methods, such as ARSER, JTK_CYCLE, and Lomb-Scargle for the detection of a particular rhythm. The ARSER method was used to determine the period of a given oscillation (58). The analysis considered a period (τ) ranging from 4 to 48 h and significance at *p* < 0.05. Time was considered as a fixed effect factor, including a linear component (time) and a periodic component (2 · π · (time-ω) · τ-1), ω being the phase, and τ the period. The linear component accounts for monotonous, nonperiodic effects of time, while the periodic component accounts for the oscillations present in the variable.

For further periodic analysis, when the model assumptions were infringed, we used the RAIN analysis which does not presume symmetric waveforms (59). The analysis considered a period (τ) ranging from 8 to 32 h and significance at *p* < 0.05.

To determine the periodic behavior of the different variables, all of them were analyzed using linear mixed models applying the "lme" function of the "nlme" package https://bugs.r-project.org (Accessed July 26, 2022). The experiments and their repeats were incorporated as nested random effect factors. Time was considered as a fixed effect factor, including a linear component (ZT) and a periodic component (2 · π · (ZT-ω) · τ-1). The linear component accounts for monotonous, nonperiodic effects of time, while the periodic component accounts for the oscillations present in the variable. Parameters such as phase (ω) and period (τ) were adjusted through an optimization process comparing models using the Akaike Information Criterion. In this optimization process, the intervals included in the sampling design from 12 and up to 32 h were tested as possible periods. The phases tested correspond to the sampling design times from zero to the maximum period contemplated. Additionally, the best fitted model was compared, using the procedure described, with a null model with the same random effect factors but with only the linear component of time (ZT) as a fixed effect factor. In all cases, the assumptions of normality and homogeneity of variance of the residues were tested. The logarithmic transformation of the variables was only applied when the assumptions were not fulfilled. The proportion of the variance explained by the fixed effect factors, such as the effect of both linear and periodic time on each variable, was estimated according to Nakagawa *et al*. (60). When the violation of the assumptions could not be corrected for a certain variable, they were analyzed using the RAIN algorithm (59), for which the values of the repeats were averaged, the measurements were standardized by experiment, and the linear effect of time was discounted when necessary, before applying the RAIN algorithm. All analyzes were carried out in the R environment (R Core Team 2017. R: A language and environment for statistical computing, Vienna, Austria URL: https://www.r-project.org).

## Data availability

All data presented are contained within the manuscript.

## Supporting information

This article contains supporting information (39).

## Conflict of interests

All authors declare that they have no conflicts of interest with the contents of the article.

## Supporting information

Suppl Table 1

Suppl Table 2

Suppl Table 3

Suppl Table 4

## References

[1] Mashek D.G. (2013). Hepatic fatty acid trafficking: multiple forks in the road. Adv. Nutr..

[2] Kennedy E.P., Weisst S.B. (1956). The function of cytidine coenzymes in the biosynthesis of phospholipides. J. Biol. Chem..

[3] Li Z., Vance D.E. (2008). Phosphatidylcholine and choline homeostasis. J. Lipid Res..

[4] Wu G., Vance D.E. (2010). Choline kinase and its function. Biochem. Cell Biol..

[5] Aoyama C., Liao H., Ishidate K. (2004). Structure and function of choline kinase isoforms in mammalian cells. Prog. Lipid Res..

[6] Kent C. (2005). Regulatory enzymes of phosphatidylcholine biosynthesis: a personal perspective. Biochim. Biophys. Acta.

[7] Marcucci H., Paoletti L., Jackowski S., Banchio C. (2010). Phosphatidylcholine biosynthesis during neuronal differentiation and its role in cell fate determination. J. Biol. Chem..

[8] Pavlovic Z., Bakovic M. (2013). Regulation of phosphatidylethanolamine homeostasis—the critical role of CTP:phosphoethanolamine cytidylyltransferase (Pcyt2). Int. J. Mol. Sci..

[9] van der Veen J.N., Kennelly J.P., Wan S., Vance J.E., Vance D.E., Jacobs R.L. (2017). The critical role of phosphatidylcholine and phosphatidylethanolamine metabolism in health and disease. Biochim. Biophys. Acta. Biomembr..

[10] Gluchowski N.L., Becuwe M., Walther T.C., Farese R.V. (2017). Lipid droplets and liver disease: from basic biology to clinical implications. Nat. Rev. Gastroenterol. Hepatol..

[11] Sookoian S., Pirola C.J. (2019). Review article: shared disease mechanisms between non-alcoholic fatty liver disease and metabolic syndrome - translating knowledge from systems biology to the bedside. Aliment. Pharmacol. Ther..

[12] Lowrey P.L., Takahashi J.S. (2004). Mammalian circadian biology: elucidating genome-wide levels of temporal organization. Annu. Rev. Genomics Hum. Genet..

[13] Bell-Pedersen D., Cassone V.M., Earnest D.J., Golden S.S., Hardin P.E., Thomas T.L. (2005). Circadian rhythms from multiple oscillators: lessons from diverse organisms. Nat. Rev. Genet..

[14] Dunlap J.C., L J.J., D P.J. (2004).

[15] Lahti T.A., Partonen T., Kyyrönen P., Kauppinen T., Pukkala E. (2008). Night-time work predisposes to non-Hodgkin lymphoma. Int. J. Cancer.

[16] Lahti T., Merikanto I., Partonen T. (2012). Circadian clock disruptions and the risk of cancer. Ann. Med..

[17] Takahashi J.S., Hong H.K., Ko C.H., McDearmon E.L. (2008). The genetics of mammalian circadian order and disorder: implications for physiology and disease. Nat. Rev. Genet..

[18] Hanahan D., Weinberg R.A. (2011). Hallmarks of cancer: the next generation. Cell.

[19] Balogh J., Victor D., Asham E.H., Burroughs S.G., Boktour M., Saharia A. (2016). Hepatocellular carcinoma: a review. J. Hepatocell. Carcinoma..

[20] King D.P., Zhao Y., Sangoram A.M., Wilsbacher L.D., Tanaka M., Antoch M.P. (1997). Positional cloning of the mouse circadian clock gene. Cell.

[21] Gekakis N., Staknis D., Nguyen H.B., Davis F.C., Wilsbacner L.D., King D.P. (1998). Role of the CLOCK protein in the mammalian circadian mechanism. Science.

[22] Preitner N., Damiola F., Luis-Lopez-Molina, Zakany J., Duboule D., Albrecht U. (2002). The orphan nuclear receptor REV-ERBalpha controls circadian transcription within the positive limb of the mammalian circadian oscillator. Cell.

[23] Zhang Y., Luo X.Y., Wu D.H., Xu Y. (2015). ROR nuclear receptors: structures, related diseases, and drug discovery. Acta Pharmacol. Sin..

[24] Edgar R.S., Green E.W., Zhao Y., Van Ooijen G., Olmedo M., Qin X. (2012). Peroxiredoxins are conserved markers of circadian rhythms. Nature.

[25] Dibner C., Schibler U. (2015). Circadian timing of metabolism in animal models and humans. J. Intern. Med..

[26] Wagner P.M., Sosa Alderete L.G., Gorné L.D., Gaveglio V., Salvador G., Pasquaré S. (2019). Proliferative glioblastoma cancer cells exhibit persisting temporal control of metabolism and display differential temporal drug susceptibility in chemotherapy. Mol. Neurobiol..

[27] Guido M.E., Monjes N.M., Wagner P.M., Salvador G.A. (2022). Circadian regulation and clock-controlled mechanisms of glycerophospholipid metabolism from neuronal cells and tissues to fibroblasts. Mol. Neurobiol..

[28] Garbarino-Pico E., Valdez D.J., Contín M.A., Pasquaré S.J., Castagnet P.I., Giusto N.M. (2005). Rhythms of glycerophospholipid synthesis in retinal inner nuclear layer cells. Neurochem. Int..

[29] Guido M.E., Garbarino Pico E., Caputto B.L. (2001). Circadian regulation of phospholipid metabolism in retinal photoreceptors and ganglion cells. J. Neurochem..

[30] Garbarino-Pico E., Carpentieri A.R., Castagnet P.I., Pasquaré S.J., Giusto N.M., Caputto B.L. (2004). Synthesis of retinal ganglion cell phospholipids is under control of an endogenous circadian clock: daily variations in phospholipid-synthesizing enzyme activities. J. Neurosci. Res..

[31] Gorné L.D., Acosta-Rodríguez V.A., Pasquaré S.J., Salvador G.A., Giusto N.M., Guido M.E. (2015). The mouse liver displays daily rhythms in the metabolism of phospholipids and in the activity of lipid synthesizing enzymes. Chronobiol. Int..

[32] Acosta-Rodríguez V.A., Márquez S., Salvador G.A., Pasquaré S.J., Gorné L.D., Garbarino-Pico E. (2013). Daily rhythms of glycerophospholipid synthesis in fibroblast cultures involve differential enzyme contributions. J. Lipid Res..

[33] Gréchez-Cassiau A., Feillet C., Guérin S., Delaunay F. (2015). The hepatic circadian clock regulates the choline kinase α gene through the BMAL1-REV-ERBα axis. Chronobiol. Int..

[34] Froy O. (2010). Metabolism and circadian rhythms--implications for obesity. Endocr. Rev..

[35] Green C.B., Takahashi J.S., Bass J. (2008). The meter of metabolism. Cell.

[36] Maury E., Ramsey K.M., Bass J. (2010). Circadian rhythms and metabolic syndrome: from experimental genetics to human disease. Circ. Res..

[37] Sookoian S., Gemma C., Gianotti T.F., Burgueño A., Castaño G., Pirola C.J. (2008). Genetic variants of Clock transcription factor are associated with individual susceptibility to obesity. Am. J. Clin. Nutr..

[38] Wagner P.M., Prucca C.G., Velazquez F.N., Sosa Alderete L.G., Caputto B.L., Guido M.E. (2021). Temporal regulation of tumor growth in nocturnal mammals: *in vivo* studies and chemotherapeutical potential. FASEB J..

[39] Wagner P.M., Monjes N.M., Guido M.E. (2019). Chemotherapeutic effect of SR9009, a REV-ERB agonist, on the human glioblastoma T98G cells. ASN Neuro.

[40] Damato A.R., Herzog E.D. (2022). Circadian clock synchrony and chronotherapy opportunities in cancer treatment. Semin. Cell Dev. Biol..

[41] Takiguchi T., Tomita M., Matsunaga N., Nakagawa H., Koyanagi S., Ohdo S. (2007). Molecular basis for rhythmic expression of CYP3A4 in serum-shocked HepG2 cells. Pharmacogenet. Genomics..

[42] Sztolsztener K., Harasim-Symbor E., Chabowski A., Konstantynowicz-Nowicka K. (2021). The influence of dexamethasone on hepatic fatty acids metabolism and transport in human steatotic HepG2 cell line exposed to palmitate. Biochem. Biophys. Res. Commun..

[43] Hughes M.E., DiTacchio L., Hayes K.R., Vollmers C., Pulivarthy S., Baggs J.E. (2009). Harmonics of circadian gene transcription in mammals. PLoS Genet..

[44] Li Z., Agellon L.B., Allen T.M., Umeda M., Jewell L., Mason A. (2006). The ratio of phosphatidylcholine to phosphatidylethanolamine influences membrane integrity and steatohepatitis. Cell Metab.

[45] Niebergall L.J., Vance D.E. (2012). The ratio of phosphatidylcholine to phosphatidylethanolamine does not predict integrity of growing MT58 Chinese hamster ovary cells. Biochim. Biophys. Acta.

[46] Farese R.V., Walther T.C. (2009). Lipid droplets finally get a little R-E-S-P-E-C-T. Cell.

[47] Andersen L.W., Mackenhauer J., Roberts J.C., Berg K.M., Cocchi M.N., Donnino M.W. (2013). Etiology and therapeutic approach to elevated lactate levels. Mayo Clin. Proc..

[48] Heiden M.G.V., Cantley L.C., Thompson C.B. (2009). Understanding the Warburg effect: the metabolic requirements of cell proliferation. Science.

[49] Brooks G.A. (2009). Cell-cell and intracellular lactate shuttles. J. Physiol..

[50] Krishnaiah S.Y., Wu G., Altman B.J., Growe J., Rhoades S.D., Coldren F. (2017). Clock regulation of metabolites reveals coupling between transcription and metabolism. Cell Metab..

[51] Chen-Goodspeed M., Cheng C.L. (2007). Tumor suppression and circadian function. J. Biol. Rhythms..

[52] Kiessling S., Beaulieu-Laroche L., Blum I.D., Landgraf D., Welsh D.K., Storch K.F. (2017). Enhancing circadian clock function in cancer cells inhibits tumor growth. BMC Biol..

[53] Wu G., Francey L.J., Ruben M.D., Hogenesch J.B. (2021). Normalized coefficient of variation (nCV): a method to evaluate circadian clock robustness in population scale data. Bioinformatics.

[54] Eruslanov E., Kusmartsev S. (2010). Identification of ROS using oxidized DCFDA and flow-cytometry. Met. Mol. Biol..

[55] Rios M.N., Marchese N.A., Guido M.E. (2019). Expression of non-visual opsins Opn3 and Opn5 in the developing inner retinal cells of birds. Light-responses in müller glial cells. Front. Cell. Neurosci..

[56] Pfaffl M.W. (2001). A new mathematical model for relative quantification in real-time RT-PCR. Nucl. Acids Res..

[57] Chao H.C., Chen G.Y., Hsu L.C., Liao H.W., Yang S.Y., Wang S.Y. (2017). Using precursor ion scan of 184 with liquid chromatography-electrospray ionization-tandem mass spectrometry for concentration normalization in cellular lipidomic studies. Anal. Chim. Acta.

[58] Yang R., Su Z. (2010). Analyzing circadian expression data by harmonic regression based on autoregressive spectral estimation. Bioinformatics.

[59] Thaben P.F., Westermark P.O. (2014). Detecting rhythms in time series with RAIN. J. Biol. Rhythms.

[60] Johnson P.C.D. (2014). Extension of Nakagawa & Schielzeth’s R2GLMM to random slopes models. Met. Ecol. Evol..

