# Supplementary material for: “Disruption of the molecular clock severely affects lipid metabolism in a hepatocellular carcinoma cell model”
Source: J Biol Chem. 2022 Sep 30;298(11):102551. doi: 10.1016/j.jbc.2022.102551 (PMC9637785; doi:10.1016/j.jbc.2022.102551)
Supplement: Suppl Table 1 [file mmc1.docx]

**SUPPL. TABLE 1: Antibody list**

| **Antibody** | **Host** | **Catalogue** | **Dilution** |
| --- | --- | --- | --- |
| Per1 | Rabbit | Abcam, ab-3443 | 1:100 (ICC) |
| Per2 | Rabbit | Abcam, ab-180655 | 1:150 (ICC) |
| Bma1 | Rabbit | NOVUS, nb-100-2288 | 1:500 (WB) |
| Bmal1 | Mouse | Abcam, ab-119009 | 1:100 (ICC) |
| REV-ERBα | Mouse | Santa Cruz Biotechnology, sc-100910 (RS-14) | 1:100 (ICC) , 1:250 (WB) |
| Dgat2 | Mouse | Santa Cruz Biotechnology, sc-293211 (4C-1) | 1:100 (ICC) , 1:200 (WB) |
| ChoKα | Mouse | Santa Cruz Biotechnology, sc-376489 (B-8) | 1:100 (ICC) |
| CCTβ | Rabbit | Sigma, hpa-006367, at-08082 | 1:200 (ICC) |
| Lipin1 | Rabbit | Abcam, ab-92316 (EPR3725) | 1:500 (WB) |
| α-Tub | Mouse | Merck Millipore, DM1A | 1:250 (WB) |
