# Supplementary material for: “Disruption of the molecular clock severely affects lipid metabolism in a hepatocellular carcinoma cell model”
Source: J Biol Chem. 2022 Sep 30;298(11):102551. doi: 10.1016/j.jbc.2022.102551 (PMC9637785; doi:10.1016/j.jbc.2022.102551)
Supplement: Suppl Table 2 [file mmc2.docx]

**SUPPL. TABLE 2: qPCR Primer list**

| **Transcript Name** | **Accession Number** | **Forward Primer (5´ 🡪 3´)**  **Reverse Primer (5´ 🡪 3´)** | **Amplicon**  **Size (bp)** | **Tm (°C)** |
| --- | --- | --- | --- | --- |
| *Tbp* | NM_003194.5 | CGGCTGTTTAACTTCGCTTCC  GAGCATCTCCAGCACACTCTT | 129 | 60 |
| *Bmal1* | NM_001351824.2 | TCCTTCCAGTGGCCTACTAT  GCCTCATCATTACTGGGACTAC | 150 | 60 |
| *Per1* | NM_001159367.1 | CGGATTGTCTATATTTCGGAGCA  TGGGCAGTCGAGATGGTGT | 142 | 60 |
| *Rev-Erbα* | NM_021724.5 | TGGCATGGTGCTACTGTGTAAAG  ATGTTCTGCTGGATGCTCCGACG | 114 | 60 |
| *Lipin1* | NM_001261429.1 | CCAGGAAAAGAGATAAACGAAGCC  AAGGGGAAACTGGTCTCACAC | 180 | 60 |
| *ChoKα* | NM_001376221.1 | GGCCAAGATCTCATCTATTGAA  TGGTGGAAATAGGCATCAAACC | 72 | 55 |
| *Pemt* | NM_148173.2 | GGGGTTCGCTGGAACTTTC  GAGCCACTATGTAGGTGAGGG | 197 | 60 |
| *Pcyt-2* | NM_001347615.1 | GTCATCGCCGGCCTACACT  GAGTCCGCTCGTGCAGGTT | 85 | 60 |
