# Supplementary material for: “Disruption of the molecular clock severely affects lipid metabolism in a hepatocellular carcinoma cell model”
Source: J Biol Chem. 2022 Sep 30;298(11):102551. doi: 10.1016/j.jbc.2022.102551 (PMC9637785; doi:10.1016/j.jbc.2022.102551)
Supplement: Suppl Table 3 [file mmc3.docx]

**SUPPL. TABLE 3: Periodic analysis for transcripts of clock genes and enzymes involved in the glycerophospholipid biosynthesis.**

|  | A. MetaCycle analysis | | | | | B. RAIN analysis | |
| --- | --- | --- | --- | --- | --- | --- | --- |
| GENE | Period | Amplitude | Phase | r^2^ | p-value | Period | p-value |
| *Bmal1/Tbp*  (serum-synchronized) | 24.95 | 0.28 | 0.694 | 0.422 | 0.109 | 24 | 0.0003 |
| *Bmal1/Tbp*  (non-synchronized) | 24 | 0.095 | 17.116 | 0.106 | 0.326 | 24 | 0.01 |
| *Pemt/Tbp*  (serum-synchronized) | 12.71 | 0.165 | 4.778 | 0,699 | 0,021 | 12 | 0.0009 |
| *Pemt/Tbp*  (non-synchronized) | 14.12 | 0.185 | 3.709 | 0.036 | 0.393 | 12 | 0.02 |
| *ChoKα/Tbp*  (serum-synchronized) | 28.65 | 0.846 | 12.315 | -0.187 | 0.661 | 12 | 0.04 |
| ChoKα/Tbp  (non-synchronized) | 14.9 | 0.115 | 3.864 | 0,999 | 8,72E-06 | - | N.S. |

**Suppl Table 3:** Periodic analysis for transcripts of clock genes and enzymes involved in the glycerophospholipid biosynthesis. MetaCycle analysis (A) using the ARSER method and RAIN analysis (B) were performed for periodic analysis and rhythmic parameters. p<0.05 indicates a significant effect for the fit of the experimental data with respect to the theoretical curve. N.S.: non-significant. See methods for further detail.
