# Supplementary material for: “Disruption of the molecular clock severely affects lipid metabolism in a hepatocellular carcinoma cell model”
Source: J Biol Chem. 2022 Sep 30;298(11):102551. doi: 10.1016/j.jbc.2022.102551 (PMC9637785; doi:10.1016/j.jbc.2022.102551)
Supplement: Suppl Table 4 [file mmc4.docx]

| Gene (transcript or PROTEIN) | MAX value ± SEM/ time (h) | MIN value ± SEM/ time (h) | p-value |
| --- | --- | --- | --- |
| *Bmal1/Tbp* | 1,151 ± 0,0022 / (t_12_) | 0.5523 ±0.04353 / (t_30_) | ns |
| *Per1/Tbp* | 18.63 ± 1.120 / (t_6_) | 0.9056 ± 0.3247 / (t_12_) | 0.006 |
| *Rev-Erbα/Tbp* | 1.277 ± 0.2046 / (t_6_) | 0.4848 ± 0.1133 / (t_36_) | 0.02 |
| *Pcyt2/Tbp* | 1.148 ± 0.1117 / (t_6_) | 0.4779 ± 0.0716 / (t_18_) | ns |
| *ChoKα/Tbp* | 1.486 ± 0.1817 / (t_36_) | 0.5763 ± 0.0036 / (t_6_) | <0.05 |
| BMAL1 | 140 ± 8.144 / (t_12_) | 95.09 ± 2.184 / (t_30_) | <0.0001 |
| PER1 | 147.8 ± 14.14 / (t_12_) | 73.28 ± 3.817 / (t_30_) | 0.0001 |

**SUPPL. TABLE 4: Statistic analysis of** **clock and clock-controlled gene expression and LD parameters, TG and Lactate content in synchronized HepG2 cells**

|  | | | |
| --- | --- | --- | --- |
| LD parameters, TG or Lactate content | MAX value / time (h) | MIN value / time (h) | p-value |
| LDs number | 73.75 ± 9.886 / (t_12_) | 32.25 ± 5.984 / (t_6_) | 0.001 |
| LDs % area | 12.48 ± 1.42 / (t_18_) | 6.083 ± 1.04 / (t_12_) | 0.01 |
| LDs average size | 1.180 ± 0.1737 / (t_0_) | 0.5357 ± 0.0713 / (t_12_) | 0.04 |
| Triglyceride | 104.8 ± 27.66 / (t_36_) | 24.99 ± 1.3 / (t_12_) | 0.008 |
| Lactate | 136.0 ± 43.55 / (t_36_) | 37.42 ± 2.957 / (t_0_) | 0.001 |

**Suppl. Table 4.** The effect of time on clock and clock-controlled gene expression, LD parameters, and TG and Lactate content in synchronized HepG2 cells was performed by ANOVA or Kruskal-Wallis (K-W) post-hoc test when the normality of residuals was infringed. Data are mean ± SEM. In all cases significance was considered at p<0.05; ns: non-significant. Transcripts are represented in italics, proteins in capital letters.
