# Supplementary material for: “Disruption of the molecular clock severely affects lipid metabolism in a hepatocellular carcinoma cell model”
Source: J Biol Chem. 2022 Sep 30;298(11):102551. doi: 10.1016/j.jbc.2022.102551 (PMC9637785; doi:10.1016/j.jbc.2022.102551)
Supplement: Supplementary Figure Legends [file mmc5.docx]

**Supplementary Figure Legends**

**Suppl. Fig. 1. Temporal organization and cell synchronization for the study of circadian rhythms.** A) The molecular clock: transcriptional-translational feedback loop (TTFL). The primary loop comprises positive (Bmal1 and Clock) and negative (Per and Cry) elements that bind to E-Box elements (violet boxes), activating or suppressing the transcription, respectively, as shown in the yellow panel on the left. A secondary loop involving the nuclear receptors RORα and REV-ERBα that regulate transcription activity (activating or repressing, respectively) via RORE sequences (orange boxes) is shown in the green panel on the right. **B**) Illustrative scheme of the synchronization protocol used in cell cultures. Colored boxes (serum-responsive elements, SRE; cAMP-responsive element, CRE; and glucocorticoid-responsive element, GRE) correspond to regulatory sequences of Per1/2 promoters involved in early transcriptional response. GRE in particular is activated by the glucocorticoid analogue dexamethasone (DEX), which is used to synchronize the molecular clock of all cells presents in the culture plate (each one is represented as an individual wave in the scheme) by a 1 h shock (blue light box). After synchronization cells were maintained under proliferative conditions (yellow boxes, 5 % of fetal bovine serum, 5 % FBS) along different times in order to evaluate temporal effects on metabolic and molecular parameters (represented as a black wave). **C**) Cell cycle phases of proliferating HepG2 cells after synchronization. The percentage of cells in each phase maintained similar levels throughout the 24 h evaluated post-synchronization, showing no significant differences (Sub-G0: p=0.6; G0-G1: p=0.6; G2-M: p=0.3; and S: p=0.7 by ANOVA with Kruskal Wallis test).

**Suppl. Fig. 2: Clock and clock-controlled gene expression in serum-shock synchronized and non-synchronized HepG2 cell cultures.** Cultures of HepG2 cells were synchronized by a 2-h horse serum shock (50%) and then collected at different times for determination of clock and clock-controlled/lipid enzyme gene mRNA expression. Non-synchronized cells maintained with 5% FBS-DMEM were subject to fresh medium exchange at time 0, and then collected at 6-h intervals for 48 h as control samples. A-C. Significant oscillations were observed in expression of *Bmal*1 mRNA with a period of 24 h (A, p=0,0003 by RAIN analysis) and in levels of *Pemt* (B) and *ChoKα* (C) mRNAs with a period of 12 h in serum-synchronized cultures (full lines) (p=0,0009 and p= 0.035 by RAIN analysis, respectively). Non-synchronized cells (dashed lines) that only were subject to fresh medium exchange at time 0 exhibited a periodic oscillation of 24 for *Bmal*1 (A, p=0,01 by RAIN analysis) and 12 h for *Pemt* (B, p=0.02 by RAIN analysis) mRNA expression with low amplitude whereas no time-related differences were seen in *ChoKα* levels along the 42 h examined. See Suppl. Table 3 for further information

**Suppl. Fig. 3. Disruption of Bmal1 and downstream effects in HepG2 cells (B-D cells**). Levels of Bmal1 protein showed a slight decrease (**A**, p=0.2 by t-test, non-significant) whereas at the protein level, the clock controlled genes PER2 and REV-ERB exhibited a significant decrease (**B**, p<0.0001, **C**, p=0.002, by t-test respectively) in B-D cells compared to B-WT cells (controls). Protein levels were determined by ICC in both populations (B-D and B-WT cells) as described in Methods. Further effects were observed at the mRNA level by qPCR in downstream genes as *Rev-Erb* (**D**), which displayed a significant increase in B-D cells as compared to B-WT cells across time post-synchronization (p=0.04 by ANOVA with K-W test for B-WT and B-D cells) with a higher amplitude and periods shorter than 24 h (see Table 2A for further detail). **p<0.01; ***p<0.001; ****p<0.0001.
